# ROS promote epigenetic remodeling and cardiac dysfunction in offspring following maternal engineered nanomaterial (ENM) exposure

**DOI:** 10.1186/s12989-019-0310-8

**Published:** 2019-06-18

**Authors:** Amina Kunovac, Quincy A. Hathaway, Mark V. Pinti, William T. Goldsmith, Andrya J. Durr, Garrett K. Fink, Timothy R. Nurkiewicz, John M. Hollander

**Affiliations:** 1 Division of Exercise Physiology, West Virginia University School of Medicine, PO Box 9227, 1 Medical Center Drive, Morgantown, WV 26506 USA; 2 Mitochondria, Metabolism & Bioenergetics Working Group, West Virginia University School of Medicine, Morgantown, WV USA; 3 West Virginia University School of Pharmacy, Morgantown, WV USA; 4 Center for Inhalation Toxicology (iTOX), West Virginia University School of Medicine, Morgantown, WV USA; 5 Department of Physiology, Pharmacology, Morgantown, WV USA

**Keywords:** Inhalation, Mitochondria, Bioenergetics, Methylation, GPx4, Hydrogen peroxide

## Abstract

**Background:**

Nano-titanium dioxide (nano-TiO_2_) is amongst the most widely utilized engineered nanomaterials (ENMs). However, little is known regarding the consequences maternal ENM inhalation exposure has on growing progeny during gestation. ENM inhalation exposure has been reported to decrease mitochondrial bioenergetics and cardiac function, though the mechanisms responsible are poorly understood. Reactive oxygen species (ROS) are increased as a result of ENM inhalation exposure, but it is unclear whether they impact fetal reprogramming. The purpose of this study was to determine whether maternal ENM inhalation exposure influences progeny cardiac development and epigenomic remodeling.

**Results:**

Pregnant FVB dams were exposed to nano-TiO_2_ aerosols with a mass concentration of 12.09 ± 0.26 mg/m^3^ starting at gestational day five (GD 5), for 6 h over 6 non-consecutive days. Aerosol size distribution measurements indicated an aerodynamic count median diameter (CMD) of 156 nm with a geometric standard deviation (GSD) of 1.70. Echocardiographic imaging was used to assess cardiac function in maternal, fetal (GD 15), and young adult (11 weeks) animals. Electron transport chain (ETC) complex activities, mitochondrial size, complexity, and respiration were evaluated, along with 5-methylcytosine, Dnmt1 protein expression, and Hif1α activity. Cardiac functional analyses revealed a 43% increase in left ventricular mass and 25% decrease in cardiac output (fetal), with an 18% decrease in fractional shortening (young adult). In fetal pups, hydrogen peroxide (H_2_O_2_) levels were significantly increased (~ 10 fold) with a subsequent decrease in expression of the antioxidant enzyme, phospholipid hydroperoxide glutathione peroxidase (GPx4). ETC complex activity IV was decreased by 68 and 46% in fetal and young adult cardiac mitochondria, respectively. DNA methylation was significantly increased in fetal pups following exposure, along with increased Hif1α activity and Dnmt1 protein expression. Mitochondrial ultrastructure, including increased size, was observed at both fetal and young adult stages following maternal exposure.

**Conclusions:**

Maternal inhalation exposure to nano-TiO_2_ results in adverse effects on cardiac function that are associated with increased H_2_O_2_ levels and dysregulation of the Hif1α/Dnmt1 regulatory axis in fetal offspring. Our findings suggest a distinct interplay between ROS and epigenetic remodeling that leads to sustained cardiac contractile dysfunction in growing and young adult offspring following maternal ENM inhalation exposure.

**Electronic supplementary material:**

The online version of this article (10.1186/s12989-019-0310-8) contains supplementary material, which is available to authorized users.

## Background

An elevated risk of postnatal cardiovascular disease has been imputed to a toxic gestational environment, when the fetus is at a critical point of development [1, 2]. The adverse effects of maternal prenatal disease and environment on future progeny were proposed as early as the 1990’s with Barker’s contribution of the Developmental Origins of Health and Disease hypothesis [3, 4]. Recent studies have focused on the consequences of a baleful gestational environment, which include endocrine disruptors, toxic metals, and the subsequent increase in progeny developing cardiovascular, cancer, reproductive, immunological, and neurological diseases [5–9]. However, investigation into organ dysfunction and the molecular consequences of maternal inhalation exposure to engineered nanomaterial (ENM) on developing offspring is lacking.

Titanium dioxide (TiO_2_) is one of the most widely used ENM, being incorporated into toothpaste, cosmetic products, food, paint, and in clinical settings for drug delivery due to its photocatalytic capabilities [10]. Although TiO_2_ has provided a wealth of benefits in these applications, the potential for adverse effects on manufacturers, consumers, and the environment raise safety concerns that warrant elucidation. Nano-TiO_2_ exposure has been shown to have detrimental effects on mitochondrial bioenergetics and cardiovascular function, which are associated with increased levels of reactive oxygen species (ROS) [11]. Mitochondrial and cardiac dysfunction are often related due in part to the cardiomyocytes’ dependence on mitochondrial ATP generation necessary for maintaining contractile function. Mitochondria are a primary target of oxidant stress due to ROS generation that arises from the electron transport chain (ETC), which can lead to peroxidation of biomembranes and impairment of ATP production [12]. As a result, antioxidant defenses are critical for mitochondrial functional preservation.

Using transcriptomics, we had previously found that maternal nano-TiO_2_ inhalation exposure induces epigenetic remodeling in offspring through histone modifications [13]. Interestingly, an antioxidant defense protein, phospholipid hydroperoxide glutathione peroxidase (GPx4), was the most significantly downregulated transcript (~ 9 fold) following exposure, when evaluating proteins that are involved in mitochondrial functional processes (PRJNA513051). GPx4 is an antioxidant enzyme that is a primary defense mechanism against oxidation of mitochondrial biomembranes. We have previously reported that overexpression of a mitochondrially-targeted GPx4, also known as mPHGPX, in a transgenic mouse model, was capable of ameliorating H_2_O_2_ levels and improving mitochondrial and cardiac function in an acute nano-TiO_2_ inhalation exposure model [14], suggesting that it may be of particular relevance during ENM exposure.

The adverse health effects of gestational ENM exposure on developing offspring have among others been attributed to epigenetic alterations [11, 13]. DNA methylation of cytosine, forming 5-methylcytosine (5mC), was one of the first epigenetic modifications identified and it predominantly occurs at 5-C-phosphate-G-3′ (CpG sites). The maintenance DNA methyltransferase, DNMT 1, is responsible for mediating epigenetic memory by propagating the initial signal, whereas DNMT 3A/3B are responsible for de novo methylation [15]. An increase in 5mC at regulatory regions often results in gene repression and silencing [16]. The cell alters CpG methylation in an attempt to respond to environmental factors [17]. During maternal ENM exposure, toxic metals can interact with the uterine environment and cross the placental barrier causing direct fetal exposure or indirectly eliciting a maternal immune response [1, 18]. Additionally, a direct effect on the fetus may be occurring as a result of changes in maternal-fetal hemodynamics following maternal ENM inhalation exposure [19]. ENM exposure during gestation may affect fetal cardiac methylation transiently or permanently, resulting in epigenetic reprogramming.

Previous studies have reported cardiovascular changes and epigenetic alterations in fetal-stage offspring of rats exposed to inhaled nano-TiO_2_ during gestation, though the mechanisms remain elusive [11, 13]. We hypothesized that maternal nano-TiO_2_ exposure during gestation may evoke epigenetic remodeling in the fetus initiated through oxidative stress, diminishing cardiac bioenergetics and contractile function. In the current study, we utilized a mouse model of maternal ENM inhalation exposure to explicate the interrelation between ROS (H_2_O_2_) and pathogenesis to cardiac bioenergetic and contractile dysfunction at the acute stage (fetal) and chronic stage (young adult) in progeny. Our study is the first to implement a mouse model in the molecular examination of cardiac alterations elicited by maternal ENM inhalation exposure and the findings suggests that the persistent deleterious consequences observed at the fetal stage may involve sustained epigenetic reprogramming in the heart.

## Methods

### Animal model

The West Virginia University Animal Care and Use Committee approved all animal studies which conformed to the most current National Institutes of Health (NIH) Guidelines for the Care and Use of Laboratory Animals manual. Friend Virus B NIH Jackson (FVB/NJ) mice (32 females, 12 males at 7 weeks) were purchased from Jackson Laboratory (Bar Harbor, ME). Because FVB mice have prominent pronuclei and reliably large litter sizes, this strain is useful in creating artificial models that are capable of overexpressing or knocking out specific genes, which could be useful in future investigations [20]. Male and female FVB mice were housed in the West Virginia University Health Sciences Center Animal Facility and given access to a rodent diet and water ad libitum. Before mating, mice were acclimated for a minimum of 48 h. Identification of the vaginal plug was used to verify pregnancy (~ 5 days) before the pregnant dams were placed randomly into either the Sham (15 pregnant dams) or nano-TiO_2_ exposure (11 pregnant dams) group at approximately gestational day 5 (Sham = GD 4.4, nano-TiO_2_ = GD 4.7) (6 exposure times). Echocardiographic assessments were performed on Sham and nano-TiO_2_ exposed pregnant dams, as well as in the fetal (Sham = GD 14.4, nano-TiO_2_ = GD 13.8) and young adult (Sham = 10.6 weeks, nano-TiO_2_ = 10.5 weeks) offspring. Pregnant dams were euthanized (8 Sham and 6 nano-TiO_2_ exposed), and the pups were removed from the uterus of the Sham and nano-TiO_2_ exposed mothers. On average, pups and maternal tissue, were harvested at gestational days 15.4 (average of 9 pups per mom) and 14.8 (average of 10 pups per mom) from Sham and nano-TiO_2_ dams, respectively. The specific gestational days may vary by + 1 due to plug checks being administered every 24 h. Each fetal sample contained all of the pooled tissue from one individual mother separated by heart, lung, and liver. Mothers (7 Sham and 5 nano-TiO_2_ exposed) belonging to the portion of the cohort to be used for the young adult study were placed back in their individual cages after echocardiographic assessment. The offspring were born with the singly-housed mothers. Lactation was allowed to occur without intervention and offspring were weaned at 23.9 (Sham) and 22 (nano-TiO_2_) days, housed with offspring from the same mother, and separated by male and female, with no more than 5 animals per cage. Young adult offspring were euthanized, on average, at 10.8 weeks (Sham) and 10.7 weeks (nano-TiO_2_), followed by tissue collection. One, randomly selected young adult was assessed from each mother (7 Sham and 5 nano-TiO_2_ exposed) for both groups, with 4 females, 3 males in the Sham group and 2 females, 3 males in the nano-TiO_2_ group. The number of samples used per study may vary due to limitations in variability of heart size between fetuses, resulting in lower amounts of protein content.

### Engineered nanomaterial inhalation exposure

**Fig. 1:**
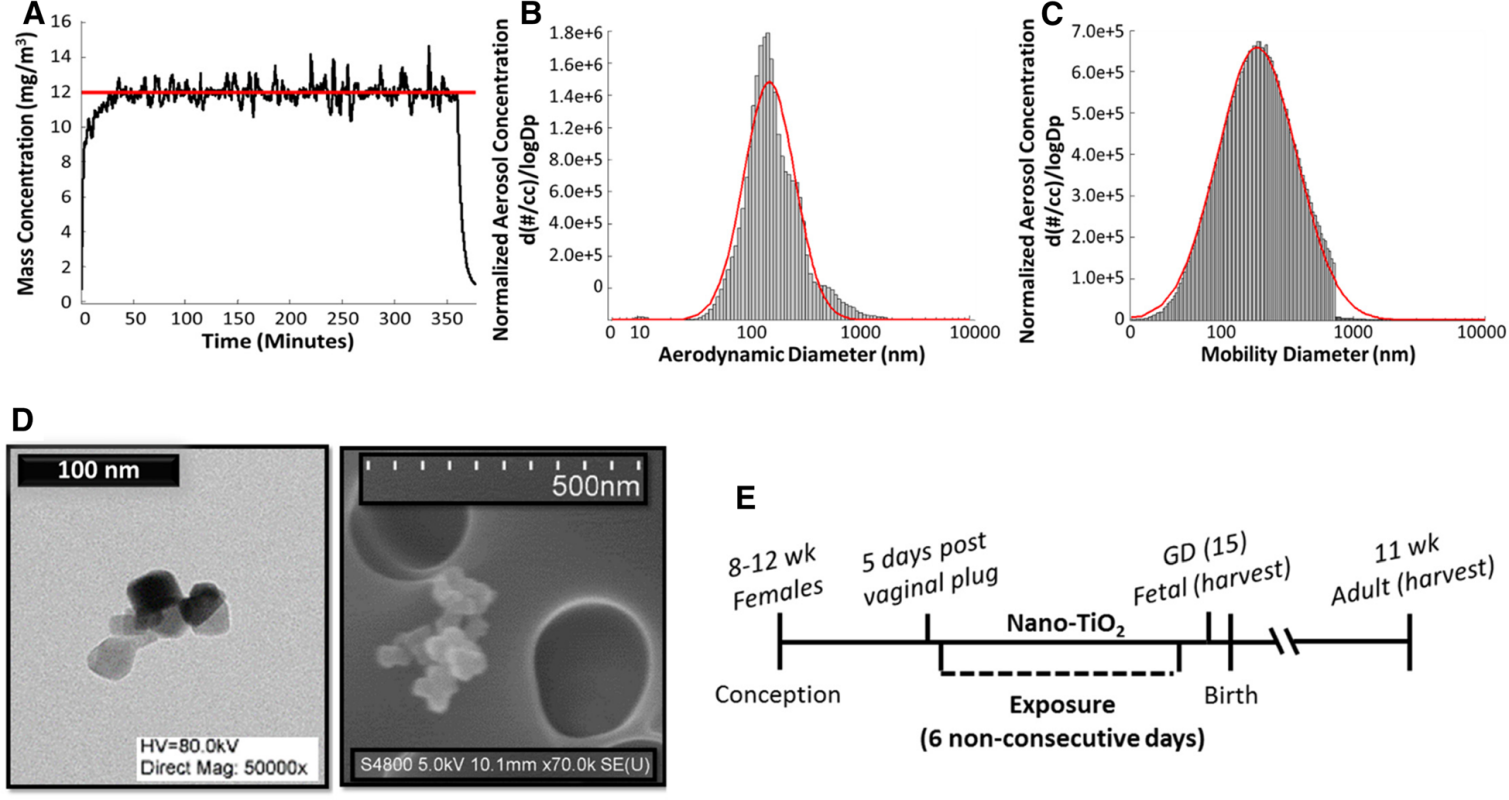
Maternal nano-TiO_2_ inhalation exposure paradigm. **a** Real-time aerosol mass concentration measurements of engineered nano-TiO_2_ during a typical maternal exposure with the target concentration indicated by the red line (12 mg/m^3^). **b** Aerodynamic diameter of nano-TiO_2_ (CMD = 156 nm) measured by high resolution electric low-pressure impactor (ELPI+). **c** Diameter of nano-TiO_2_ (CMD = 184 nm) measured by combining scanning mobility particle sizer (SMPS) and aerodynamic particle sizer (APS) measurements. Red line indicates log-normal fit. **d** Transmission and scanning electron micrographs of nano-TiO_2_ aerosolized particles. **e** A timeline of the study. CMD = Count Median Diameter. All data are presented as the mean ± standard error of the mean (SEM)

Nano-TiO_2_ P25 powder containing anatase (80%) and rutile (20%) TiO_2_ was purchased from Evonik (Aeroxide TiO_2_, Parsippany, NJ) and prepared by drying, sieving, and storing as previously described [21, 22] . The primary particle size (21 nm), the specific surface area (48.08 m^2^/g), and the Zeta potential (− 56.6 mV) have been previously reported [14, 22, 23]. The use of the nanoparticle aerosol generator for rodent inhalation exposure has been previously described [24]. Aerosol characterization of nano-TiO_2_ data are shown in Fig. 1. To model the lung burden of nano-TiO_2_ exposure during manufacturing, a target aerosol mass concentration of 12 mg/m^3^ of engineered nano-TiO_2_ for a period of 360 min per day for 6 non-consecutive days was chosen. Figure 1a shows the mass concentration measurements over a typical exposure day; final measurements indicated a daily 360-min equivalent average mass concentration of 12.09 ± 0.26 mg/m^3^. A high-resolution electrical low-pressure impactor (ELPI+; Dekati, Tampere, Finland), a scanning particle mobility sizer (SMPS 3938; TSI Inc., St. Paul, MN), and an aerodynamic particle sizer (APS 3321; TSI Inc., St. Paul, MN) were used to measure the size of the nano-TiO_2_ aerosols. A log-normal fit of the data from the ELPI+ indicated an aerodynamic aerosol size distribution with a CMD of 156 nm and GSD of 1.70 (Fig. 1b). A log-normal fit of the combined data from the SMPS and APS indicated a sized distribution with a CMD of 184 nm and GSD of 2.01 (Fig. 1c). Scanning and transmission electron micrographs (SEM and TEM) of nano-TiO_2_ aerosolized particles, sampled from the exposure chamber, are shown in Fig. 1d. The dose required to match the appropriate lung deposition was calculated based on previously described mouse methodology [14]. The formula *D* = *F x V x C x T*, where F is the deposition fraction (10%), V is the minute ventilation based on body weight (36.4 ml) [25], C is the mass concentration (12.09 mg/m^3^) and T is the exposure duration (360 min), was employed [22, 26]. This resulted in a daily deposited nano-TiO_2_ lung dose of 15.85 μg (total six exposure dose =95.10 μg). Bedding material soaked with water was used in the exposure chamber to maintain a comfortable humidity during the exposure. Control animals were exposed to HEPA filtered air only with similar chamber conditions in terms of temperature and humidity. The last exposure was conducted 48 h prior to sacrifice and tissue harvesting. A timeline for the study can be seen in Fig. 1e.

### Echocardiography

Echocardiographic assessments were carried out as previously described [11, 14, 27, 28], in both nano-TiO_2_ exposed (*n* = 11) and Sham filtered-air exposed pregnant dams (*n* = 15) as well as in the pups at both the fetal (GD 14) and young adult (11 weeks) time points. Echocardiography was analyzed for one fetal pup (first pup in either the right or left horn (*n* = 15 Sham, *n* = 11 nano-TiO_2_) and one young adult (*n* = 7 Sham, *n* = 5 nano-TiO_2_) from each mother that was exposed during gestation. Each mouse was anesthetized with inhalant isoflurane, which was then maintained at or below 1% isoflurane to ensure a physiologically relevant heart rate throughout the experiment and reduce the anesthetic-induced effects on cardiac function. Motion mode (M-mode) echocardiographic and Pulse Wave Doppler images were obtained using the Vevo 2100 Imaging System (Visual Sonics, Toronto, Canada).

In maternal and young adult animals, grayscale M-mode parasternal short-axis images at the mid-papillary level of the left ventricle (LV) were used for conventional echocardiographic analysis. In M-mode, interventricular septal, inner, and posterior wall measurements were taken to determine LV thickness on adjacent end-systolic and diastolic peaks in relation to LV trace analysis. Mitral valve Doppler echocardiography was used to asses diastolic function by taking measurements of E- and A- wave velocity, deceleration time, intraventricular relaxation/contraction time, E-wave-to-A-wave ratio, mitral valve area, etc. In fetal pups, cardiac function was also assessed using M-mode and B-mode stress strain by visualizing individual pups in the uterine horn [29]. Pulse Wave Doppler echocardiography (Vascular Package) was also used to asses umbilical and uterine flow in pregnant dams using measures of peak systolic velocity, end diastolic velocity, and velocity-time integral [30]. These measurements were calculated over three cardiac cycles and averaged. All echocardiographic measurements were acquired by one analyst blinded to the animal exposure group.

### Speckle-tracking-based strain

Speckle-tracking-based strain assessments were completed using short and long axis B-mode images as previously described by our laboratory [11, 27, 28]. During each cardiac cycle, measures of strain (total deformation length divided by the original length of a segment, strain rate, displacement length, and velocity) were obtained [31, 32]. Endocardium walls were traced and analyzed for three cardiac cycles using a speckle-tracking algorithm in Visual Sonics VevoStrain software (Toronto, Canada). Time-to-peak analysis for curvilinear data, allowing strain and strain rate to be determined, were generated. For maternal and young adult cohorts, speckle-tracking was performed on both long and short axis B-mode images, while fetal groups were only assessed in the short axis. All speckle-tracking-based strain analyses were completed by the same blinded analyst.

### Fetal cardiomyocyte isolation

Pregnant dams were euthanized one day after echocardiographic assessment, and pups were removed at GD 15 from the nano-TiO_2_ exposed and Sham mouse uteri. Maternal and fetal hearts were removed through a midsagittal cut in the thoracic cavity and fetal hearts were pooled (one fetal heart from each exposed mother; Sham = 6 dams, nano-TiO_2_ = 5 dams) as previously described [11, 33]. The hearts were chopped up and initially washed with 1X ADS buffer (0.1 M NaCl, 1.2 mM NaH_2_PO_4_, 0.8 mM MgSO_4_, 5.4 mM KCl, and 5 mM glucose at pH 7.4). Digestive solution, comprised of 2 mg pancreatin (Sigma Aldrich, St. Louis, MO) and 2 mg of collagen type II (Worthington Biochemical, Lakewood, NJ), mixed with 2 mL of physiological 1X ADS buffer per heart digested, was used to isolate cells. The supernatant was removed from the tissue debris and centrifuged at 180 x g for 7 min after each saved digestion. The supernatant was discarded, and 2 ml of newborn calf serum were added to the cell suspension and stored in a 37 °C incubator.

A Percoll gradient was made in a 15 mL conical tube for separation of cardiomyocytes from other cells of the heart as previously described [11, 33]. The Percoll gradient (density = 1.130 g/ml) was made of two layers: clear (top, density = 1.059 g/ml) and red (bottom, density = 1.082 g/ml). After all cell collection steps, cells were centrifuged at 180 x g and resuspended in 2 ml of 1X ADS buffer, per five hearts. The 2 ml of cells were added to the top of the Percoll gradient and centrifuged at 1620 x g for 30 min, with 9-min acceleration and deceleration time. The non-myocardial mesenchymal cells were aspirated, and the cardiac cells were extracted from the middle layer, washed with newborn calf serum, and placed in plating media (with fetal bovine serum). Two hours after placing in plating media, cells were changed to maintenance media (no fetal bovine serum). Cells were counted using a hemocytometer.

### Mitochondrial isolation

Young adult mice (one from each exposed mother; Sham = 7, nano-TiO_2_ = 5)) were sacrificed, and hearts were excised through a midsagittal cut in the thoracic cavity. Using differential centrifugation, isolation of mitochondrial, cytosolic, and nuclear fractions was achieved. Subsarcolemmal and interfibrillar mitochondrial subpopulations were isolated as previously described [34] with modifications by our laboratory [35–37]. Due to the limited amount of subpopulation-specific mitochondria, the two subpopulations were combined. The isolated mitochondria were then resuspended in KME buffer (100 mM KCl, 50 mM MOPS and 0.5 mM EGTA pH 7.4). Protein concentrations were determined using the Bradford method with bovine serum albumin as a standard [38].

### Mitochondrial respiration

Freshly isolated mitochondria (from young adults) were used to analyze state 3 and state 4 respiration as previously described [39, 40], with modifications by our laboratory [24]. Once isolated, mitochondria were resuspended in KME buffer and protein concentrations were determined. Mitochondrial protein was added to respiration buffer (80 mM KCl, 50 mM MOPS, 1 mmol/l EGTA, 5 mmol/l KH_2_PO_4_ and 1 mg/ml BSA) and placed into a respiration chamber connected to a multi-unit (8 channel) Oxytherm Peltier Electrode apparatus (Hansatech Instruments, Norfolk, England). Glutamate (5 mM) and malate (5 mM) were used to initiate maximal complex I-mediated respiration. State 3 (250 mM ADP) and state 4 (ADP-limited) respiration data were expressed as nmol of oxygen consumed/min/mg protein.

### Cellular and tissue bioenergetics

Fetal isolated cardiomyocytes were plated on F96 V3 cell culture microplates and the Seahorse XF96 was used for analysis (Agilent Technologies, Santa Clara, CA) [11, 41]. The pooled samples of fetal cardiomyocytes from the pups of Sham (one pup from each of 6 dams, *n* = 1) and nano-TiO_2_-exposed (one pup from each of 5 dams, *n* = 1) females were analyzed 5 h after plating and normalized to cell number. With the pooled samples, 5–6 replicates per animal group were plated. Isolated cardiac tissue from the young adult offspring were plated on F24 V7-PS cell culture microplates. Oxygen consumption rate, extracellular acidification rate, and proton production rate were measured using oligomycin, carbonyl cyanide-*p*-trifluoromethoxyphenylhydrazone, antimycin A, and rotenone as previously described [42]. Mitochondrial respiration measurements included ATP production, proton leak, basal respiration, maximal respiration, and spare capacity.

### Mitochondrial size and internal complexity

Size and internal complexity were analyzed in mitochondria isolated from young adult offspring hearts using flow cytometry, as previously described [24, 37, 39, 43, 44]**.** Isolated mitochondria were loaded with the fluorescent probe Mitotracker Deep Red 633 (Invitrogen) for assessment of mitochondrial size and internal complexity, utilizing the forward scatter (FSC), side scatter (SSC), and Sphero AccuCount Blank Particles, 2.0 μm (Spherotech Inc., Lake Forest, IL) sizing beads. Sizing beads allowed for absolute measurement of mitochondrial size through FSC. All flow cytometric measures were captured using the LSRFortessa (BD Biosciences, Franklin Lakes, NJ) at the West Virginia University Flow Cytometry & Single Cell Core Facility. To process data, FCS Express Flow Research Edition (De Novo Software, Glendale, CA) was implemented.

### Electron transport chain (ETC) complex activities

ETC Complex activities (I, III, IV, V) were measured in maternal, fetal, and young adult hearts, placenta, and fetal and young adult liver and lung as previously described [11, 35, 39, 41]. For the maternal, fetal, and young adult analyses, whole tissue was homogenized using the Polytron PowerGen 500 S1 tissue homogenizer (Fisher Scientific, Hampton, NH) in RIPA buffer (Life Technologies, Grand Island, NY). The Bradford assay provided normalization of samples by protein content [38]. Complex I (reduction of decyclubiquinone), complex III (reduction of cytochrome *c*), complex IV (oxidation of reduced cytochrome *c*), and complex V (pyruvate kinase and phosphoenolpyruvate and ATP production) activities were measured. Final values were expressed as nanomoles consumed per minute per milligram of protein, which was equal to the nanomoles of NADH oxidized per minute per milligram of protein.

### Hydrogen peroxide (H_2_O_2_) production

Cardiac H_2_O_2_ production was analyzed in maternal, fetal, and young adult hearts, placenta, and in fetal and young adult liver and lung using Amplex Red fluorescent dye in the presence of Horse Radish Peroxidase (HRP). The Amplex Red fluorescent dye reacts with H_2_O_2_ producing resorufin, a red fluorescent oxidation product. Experiments were carried out using the manufacturer’s protocol with minor modifications [14]. Cardiac protein was incubated with HRP and Amplex Red dye was added, followed by initiation of mitochondrial H_2_O_2_ production using glutamate and malate as substrates. The Flex Station 3 fluorescent plate reader (Molecular Devices, Sunnyvale, CA) was used to assess changes in fluorescence over time and allowed for quantification of H_2_O_2_ production normalized per milligram of protein. Due to its electrical neutrality providing the ability to infiltrate out of the mitochondrial membrane, measuring H_2_O_2_ as a method of assessing mitochondrial ROS production is a well-established method, along with being more quantitative than methods used to detect other ROS [45]. Additionally, assessment of H_2_O_2_ levels is particularly important due to its high reactivity towards imperative cellular targets, as compared to using other methods of determining ROS levels such as measuring superoxide, which is not as reactive [45].

### Western blot analyses

4–12% gradient Bis-Tris gels were used in SDS-PAGE as previously described [27, 35–37, 40, 46]. Protein sample concentrations were standardized with bovine serum albumin using the Bradford method [38]. The primary antibodies used included the following: GPx4 (product no.: 10005258, Cayman Chemical, Ann Arbor, MI), Hif1α (product no.: sc53546, Santa Cruz Biotechnology INC., Dallas, TX), Dnmt1 (product no.: sc271729, Santa Cruz Biotechnology), and Dnmt3b (product no.: ab2851, Abcam, Cambridge, MA). The anti-GAPDH primary antibody (product no.: ab8245, Abcam) was used to normalize protein levels for each blot. Secondary antibodies used included the following: goat anti-mouse IgG HRP conjugate (product no.: 31430; Pierce Biotechnology, Rockford, IL) and goat anti-rabbit IgG HRP conjugate (product no.: 10004301; Cayman Chemical). Pierce enhanced chemiluminescence Western blotting substrate (Pierce Biotechnology) was used to detect signal per manufacturer’s instructions. The G:Box Bioimaging system (Syngene, Frederick, MD) was used to detect signals, and data were captured using GeneSnap/GeneTools software (Syngene). Image J Software (NIH, Bethesda, MD) was used to analyze densitometry. All values were expressed as optical density with arbitrary units.

### 5-mC DNA analyses

Twenty mg of fetal and young adult tissue was cut into ~ 2 mm^3^ and the DNeasy Blood & Tissue Kit (Qiagen, Hilden, Germany) was used, per manufacturer’s instructions, to isolate total DNA. DNA methylation (5-methylcytosine; 5-mC) levels were quantified in the fetal and young adult using a 5-mC DNA ELISA kit (Catalog no: D5326: Zymo Research Corp., Irvine, CA), per manufacturer’s instructions. Briefly, 100 ng of DNA was run per well in duplicate on a 96-well plate. DNA was denatured and bound to the 96-well plates. An anti-5-Methylcytosine monoclonal antibody was used with a secondary antibody and HRP developer. Absorbance was measured using the Flex Station 3 fluorescent plate reader (Molecular Devices, Sunnyvale, CA) at 450 nm. Using a logarithmic second-order regression equation, total percent (%) methylation was determined.

### Hif1α transcription factor analyses

Hif1α transcription factor activity was assessed in maternal and fetal hearts, placenta, and fetal lung and liver tissue samples using the Hif1α Transcription Factor Assay kit (Catalog no: 10006910: Cayman Chemical, Ann Arbor, MI), per manufacturer’s instructions. Briefly, fetal nuclear extracts were run, 10 μL per well, in duplicate on a 96-well plate, with the Hif1α response element immobilized. Hif1α binds to the response element forming an active HIF transcription factor, which was detected using a Hif1α primary antibody and a secondary antibody conjugated to HRP. Colorimetric analysis was performed using the Flex Station 3 fluorescent plate reader (Molecular Devices, Sunnyvale, CA) at 450 nm. The Hif1αtranscription factor activity was normalized using the Bradford method and reported per microgram of protein [38].

### DNMT activity analyses

DNMT activity in fetal heart was measured using a colorimetric assay kit (Catalog no: P-3009: Epigentek Group Inc., Farmingdale, NY), per manufacturer’s instructions. Nuclear extracts were loaded onto a 48-well microplate with Adomet, a universal DNMT substrate, and incubated allowing the DNMT enzymes in the protein sample to transfer methyl groups from Adomet to cytosine, methylating DNA. The wells were washed, and the methylated DNA was captured using an anti-5-mC antibody, then detected using a secondary antibody and enhancer solution. Absorbance was measured using the Flex Station 3 fluorescent plate reader (Molecular Devices, Sunnyvale, CA) at a wavelength of 450 nm and DNMT activity was proportional to the optical density intensity measured. Samples were normalized using the Bradford method and reported per microgram of protein [38].

### Ex vivo micro-CT

Microcomputed tomography (micro-CT) was used to examine the anatomy of the fetal mouse circulatory system as previously described [47]. Briefly, one fetus was dissected from a nano-TiO_2_ exposed dam and fixed in 10% formalin for 24 h before being stained with Lugol (product no.: L6146: Sigma Aldrich) solution containing iodine for one week. The fetus was then taken out of the Lugol solution and transferred to a 0.1 N iodine solution (product no.: SI861: Fisher Scientific, Hampton, NH) for one week. After staining, the sample was embedded in agar to be imaged on a high-resolution micro-CT scanner. Imaging, which took approximately six hours, was done with the scanner set to a voltage of 100 kV and current at 100 μA.

### Transmission Electron microscopy

Mitochondrial ultrastructure was analyzed in four whole fetal hearts, two from each a Sham and nano-TiO_2_ exposed mother, after being processed and imaged at the WVU Electron Microscopy Histopathology and Tissue Bank. Due to their small size, the whole fetal hearts were fixed in EM Primary Fixative (3% glutaraldehyde buffered with 0.1 M Cacodylate buffer) for 48 h. After rinsing in 0.1 M Cacodylate buffer, the samples were fixed in 1% osmium tetroxide/0.8% potassium ferricyanide solution for 1.5 h, followed by an additional series of 0.1 M Cacodylate buffer washes. Samples were dehydrated through graded alcohols (50, 75, 95, 100%) and acetone, and tissue were infiltrated with a mixture of acetone/resin for one hour each at decreasing ratios (2:1, 1:1, 1:2) followed by 2 infiltrations with pure resin for an hour each under vacuum. The whole fetal hearts were embedded into flat embedding molds and cured at 70 °C overnight. 15. Samples were then cut into 85 nm sections on a LEICA UCT 9 Ultramicrotome (Leica Microsystems, Wetzler, Germany) and mounted on copper-coated 200 mesh VELCO grids (2 per sample). The grids were stained with uranyl acetate and lead citrate solutions and imaged using the JEOL 1010 TEM with side-mount AMT digital camera (JOEL, Akishima, Tokyo, Japan). Semi-quantitative analyses of mitochondrial size were processed through Fiji (NIH). Six to eight randomly selected TEM images of mitochondria per group were used to perform analyses. Mitochondria were quantified through black & white thresholding using the Shanbhag method [48], through individual tracings, color inversion, and quantification.

### Statistics

All statistical analyses were performed using GraphPad Prism SoftwareVersion 7 for Windows (GraphPad Software, La Jolla CA). To determine statistical significance between Sham and nano-TiO_2_-exposed groups, a two-tailed Student’s t-test was used. Statistical difference was defined by *P* ≤ 0.05 = *, *P* ≤ 0.01 = **, *P* ≤ 0.001 = ***. All data are presented as the mean ± the standard error (SEM) of the mean.

## Results

### Cardiac function in maternal, fetal, and young adult animals

**Fig. 2:**
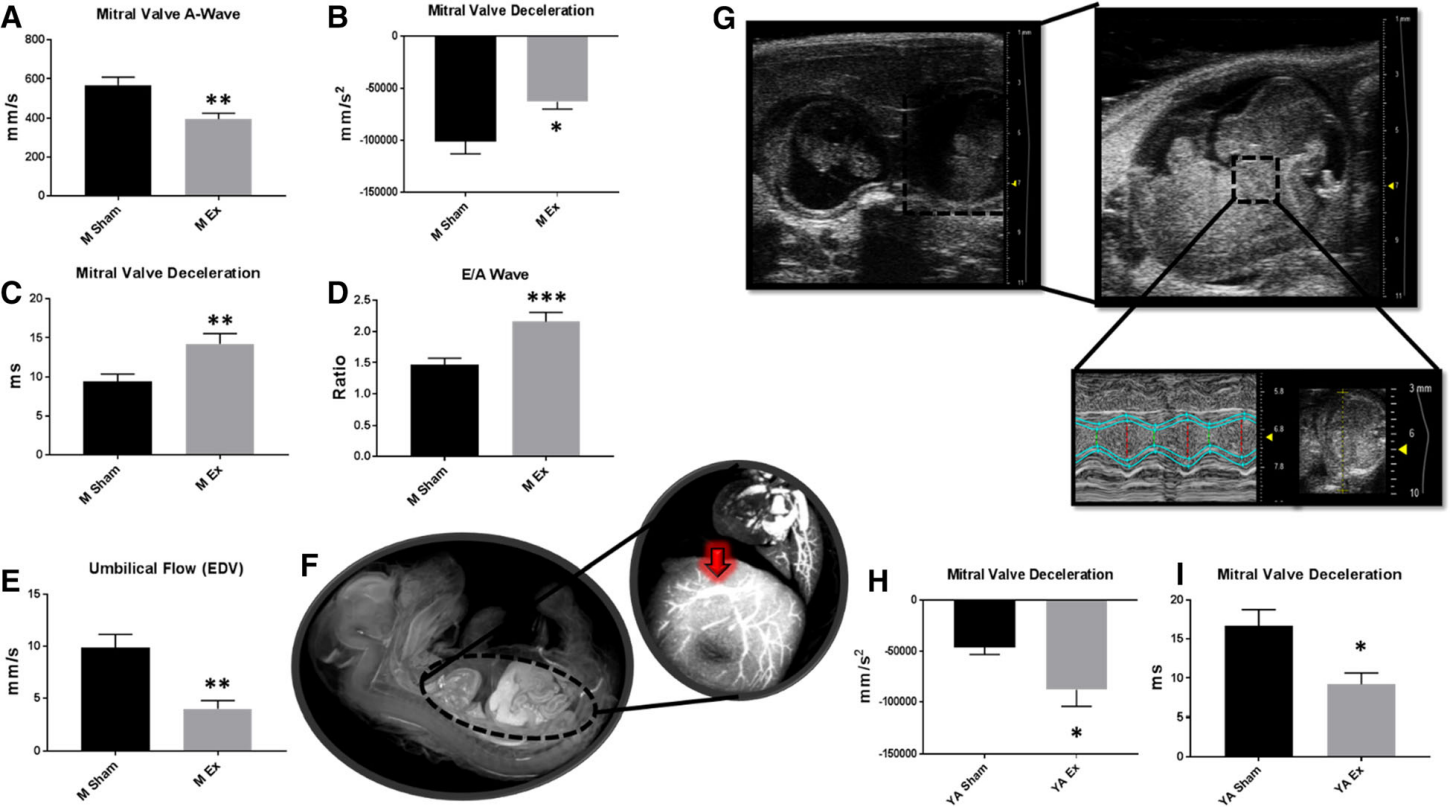
Pulse Wave Doppler assessment of Sham vs. nano-TiO_2_ exposed dams and their offspring. **a** Maternal (*n* = 15 Sham, *n* = 11 Ex) Pulse Wave Doppler-Mitral Valve imaging illustrating changes in MV-A velocity. **b** Maternal (*n* = 15 Sham, *n* = 11 Ex) Pulse Wave Doppler-Mitral Valve imaging illustrating changes in MV Deceleration speed. **c** Maternal (*n* = 15 Sham, *n* = 11 Ex) Pulse Wave Doppler-Mitral Valve imaging illustrating changes in MV Deceleration time. **d** Maternal (*n* = 15 Sham, *n* = 11 Ex) Pulse Wave Doppler-Mitral Valve imaging illustrating changes in E/A ratio. **e** Pulse Wave Doppler-Flow indices revealed a change in umbilical EDV. **f** Representative micro-CT image illustrating fetal organ complexity at GD 15 and the umbilical vein (circled in red). **g** Ultrasound image showing uterine horn containing two fetal pups, a single fetal pup, and echocardiographic gating of the fetal with a representative M-mode measurement. **h** Pulse Wave Doppler-Mitral Valve of young adult (*n* = 7 Sham, *n* = 5 Ex) following maternal exposure indicating changes in MV Deceleration speed. **i** Pulse Wave Doppler-Mitral Valve of young adult (*n* = 7 Sham, *n* = 5 Ex) following maternal exposure indicating changes in MV Deceleration time. Sham = control filtered air exposed, Ex = nano-TiO_2_ exposed, Maternal (M) = 12-week-old pregnant dams, Fetal (F) = GD (15), Young Adult (YA) = 11 weeks, MV = Mitral Valve, EDV = End Diastolic Velocity, micro-CT = micro computed tomography. All data are presented as the mean ± standard error of the mean (SEM). * = *P* ≤ 0.05, ** = *P* ≤ 0.01, *** = *P* ≤ 0.001 for Ex vs. Sham

Although nano-TiO_2_ inhalation exposure has been shown to negatively impact cardiac function in murine models [11, 13], it is not known if these effects are mimicked following maternal exposure in the fetus. In pregnant dams and fetal and young adult offspring, M-mode (Table 1) was used to assess cardiac function. Both diastolic diameter and volume were significantly decreased in the nano-TiO_2_ exposed pregnant dams as well as stroke volume and cardiac output. Pulse Wave Doppler indices revealed a significant decrease in the Mitral Valve (MV)-A velocity and a significant increase in MV deceleration - acceleration, MV deceleration time, and E/A ratio in the nano-TiO_2_ exposed mothers (Fig. 2a-d). Pulse Wave Doppler revealed no significant changes in uterine flow (Additional file 1: Table S1), but a significant decrease in umbilical end-diastolic velocity (Fig. 2e). This effect may be occurring as a result of increased vascular resistance in the in utero circulation, which has been shown to reduce placental perfusion [19] and could increase the rate of perinatal mortality [49].

**Table 1:** M-Mode Echocardiography

	Maternal		Fetal		Young Adult	
Parameter	Sham	Ex	Sham	Ex	Sham	Ex
Heart Rate (BPM)	672.05 ± 8.30	685.39 ± 7.01	134.28 ± 8.72	118.94 ± 3.40	649.49 ± 23.72	601.87 ± 65.46
Diameter;s (mm)	0.58 ± 0.05	0.59 ± 0.08	0.25 ± 0.02	0.24 ± 0.02	0.38 ± 0.05	0.73 ± 0.13*
Diameter;d (mm)	2.70 ± 0.08	2.43 ± 0.05*	0.71 ± 0.02	0.69 ± 0.03	1.95 ± 0.13	2.25 ± 0.15
Volume;s (uL)	0.64 ± 0.16	0.53 ± 0.16	0.05 ± 0.01	0.04 ± 0.01	0.14 ± 0.06	0.58 ± 0.13**
Volume;d (uL)	27.60 ± 1.92	21.15 ± 1.13**	0.85 ± 0.08	0.79 ± 0.08	12.64 ± 2.21	14.50 ± 0.58
Stroke Volume (uL)	26.96 ± 1.83	20.43 ± 1.08*	0.80 ± 0.07	0.74 ± 0.07	12.46 ± 2.17	13.92 ± 0.48
Ejection Fraction (%)	97.84 ± 0.43	96.72 ± 0.99	95.16 ± 0.84	94.79 ± 0.67	98.84 ± 0.33	94.65 ± 1.53*
Fractional Shortening (%)	78.56 ± 1.67	75.89 ± 2.95	65.68 ± 2.31	65.62 ± 1.56	80.80 ± 1.89	68.38 ± 3.14**
Cardiac Output (mL/min)	18.17 ± 1.31	13.79 ± 0.67*	0.10 ± 0.01	0.08 ± 0.01*	8.30 ± 1.68	9.35 ± 0.34
LV Mass (mg)	88.03 ± 4.89	75.53 ± 8.13	0.42 ± 0.04	0.74 ± 0.13*	83.42 ± 7.13	79.05 ± 5.59

M-mode echocardiography imaging following maternal nano-TiO_2_ inhalation exposure. Measurements were taken for a minimum of 3 consecutive systolic and diastolic peaks and troughs for maternal (*n* = 15 Sham, *n* = 11 Ex), fetal (*n* = 15 Sham, *n* = 11 Ex), and young adult (*n* = 7 Sham, *n* = 5 Ex). Sham = control filtered air exposed, Ex = nano-TiO_2_ exposed, Maternal (M) = 12 week old pregnant dams, Fetal (F) = GD (15), Young Adult (YA) = 11 weeks, Diameter;d = diastolic diameter, Diameter;s = systolic diameter, LV Mass = left ventricular mass, V;d = volume during diastole, V;s = volume during systole. All data are presented as the mean ± standard error of the mean (SEM). * = *P* ≤ 0.05, ** = *P* ≤ 0.01 for Ex vs. Sham

To better understand how maternal exposure alters fetal circulation, a micro-CT was performed in a maternal nano-TiO_2_ exposed progeny (Fig. 2f). The umbilical vein, whose diameter can be measured as way of determining vascular resistance, is also illustrated (Fig. 2f). Figure 2g shows a representative image of the uterine horn with two pups closest to the cervix, as well as an illustration of how fetal cardiac tissue was gated. In order to assess fetal cardiac function, each individual pup was visualized in the uterine horn and echocardiographic scans were performed in utero to determine the effects of maternal ENM inhalation exposure. In the fetal pups, M-mode measurements revealed a significant decrease in cardiac output and a decrease in LV mass, indicating a decrease in heart size (Table 1). Following maternal ENM inhalation exposure, young adult offspring showed a significant decrease in ejection fraction and fractional shortening compared to controls, indicating changes in LV pump (Table 1). Pulse Wave Doppler measurements were also taken at the young adult stage and indicated a significant decrease in MV deceleration - acceleration and deceleration time (Fig. 2h-i).

Speckle-tracking stress strain assessments of the pregnant dams and fetal and young adult offspring are included (Additional file 1: Tables S2–5). The maternal stress-strain measurements indicated diastolic dysfunction by an increase in long axis diastolic radial velocity and strain rate, with a significant decrease in long axis systolic radial displacement, radial velocity, and short axis circumferential strain. Short axis systolic radial strain rate and circumferential displacement were both significantly decreased in the fetal offspring of ENM-exposed dams. Short axis diastolic radial displacement was significantly decreased in the young adult and diastolic longitudinal displacement was significantly increased.

### Mitochondrial bioenergetics

**Fig. 3:**
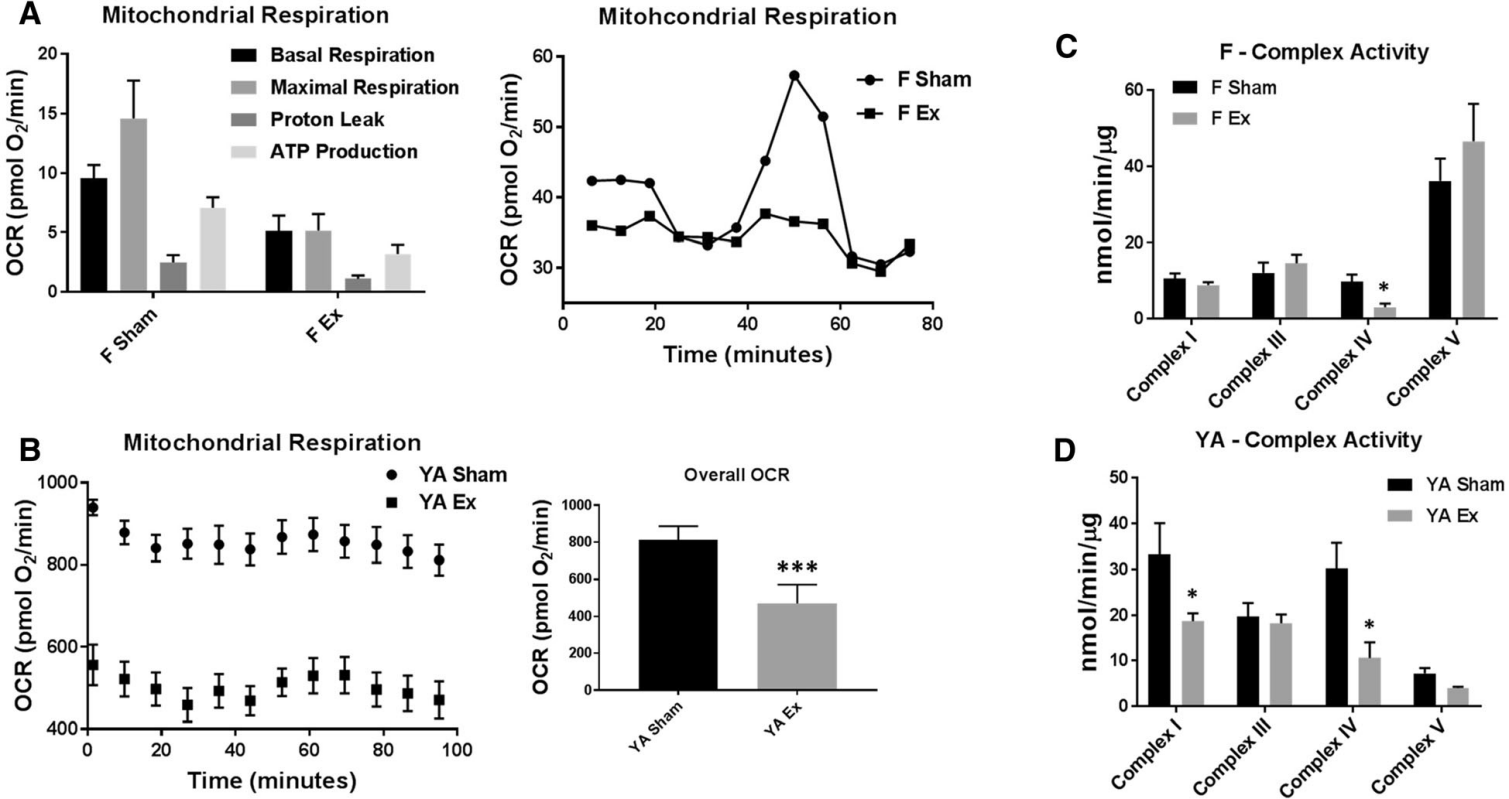
Mitochondrial bioenergetics from fetal and young adult offspring following maternal nano-TiO_2_ inhalation exposure. **a** Seahorse analyses of cardiomyocytes at the fetal stage (*n* = 1 Sham (one fetal heart from each of 6 dams pooled), *n* = 1 Ex (one fetal heart from each of 5 dams pooled) plated as 5–6 replicates) indicating changes in ATP production and basal and maximal respiration. **b** Seahorse analysis of young adult (*n* = 7 Sham, *n* = 5 Ex) animals demonstrating a significant change in overall OCR. **c** ETC Complex Activities for complexes I, III, IV, V (ATP synthase) in fetal offspring (*n* = 6 Sham, *n* = 5 Ex). **d** ETC Complex Activities for complexes I, III, IV, V (ATP synthase) in young adult offspring (*n* = 7 Sham, *n* = 5 Ex). Sham = control filtered air exposed, Ex = nano-TiO_2_ exposed, Maternal (M) = 12-week-old pregnant dams, Fetal (F) = GD (15), Young Adult (YA) = 11 weeks, OCR = Oxygen Consumption Rate, ETC = Electron Transport Chain. Complex V is measured as nmol/min/mg of tissue. All data are presented as the mean ± standard error of the mean (SEM). * = *P* ≤ 0.05, ** = *P* ≤ 0.01, *** = *P* ≤ 0.001 for Ex vs. Sham

In order to elucidate whether the cardiac dysfunction seen in the offspring of ENM-exposed dams was associated with dysregulation of mitochondrial bioenergetics, mitochondrial respiration was assessed in isolated cardiomyocytes of the fetal pups as well as in isolated mitochondria and whole tissue from young adult progeny. Basal and maximal respiration and ATP production were decreased in the fetal pups of nano-TiO_2_ exposed dams as compared to those of Sham-exposed dams (Fig. 3a). No changes in state 3 and state 4 respiration, using both glucose and fatty acid-mediated metabolism, were observed between the Sham- and nano-TiO_2_ exposed young adult offspring (Additional file 2: Figure S1A), though in isolated cardiac tissue from the young adult, an overall decrease in oxygen consumption rate was observed (Fig. 3b). ETC complex activities were evaluated in the fetal (Fig. 3c) and young adult (Fig. 3d) progeny heart with additional measurements made in the maternal heart, placenta, and fetal and young adult lung and liver (Additional file 2: Figure S1B-E). Interestingly, in the placenta, there was a significant decrease in complexes III, IV and V activities in the nano-TiO_2_ exposed dams. ETC complex IV activity (Fig. 3c, d) was significantly decreased in both the fetal and young adult offspring heart of ENM-exposed dams, further supporting alterations to mitochondrial bioenergetics [50].

### H_2_O_2_-mediated pathways

**Fig. 4:**
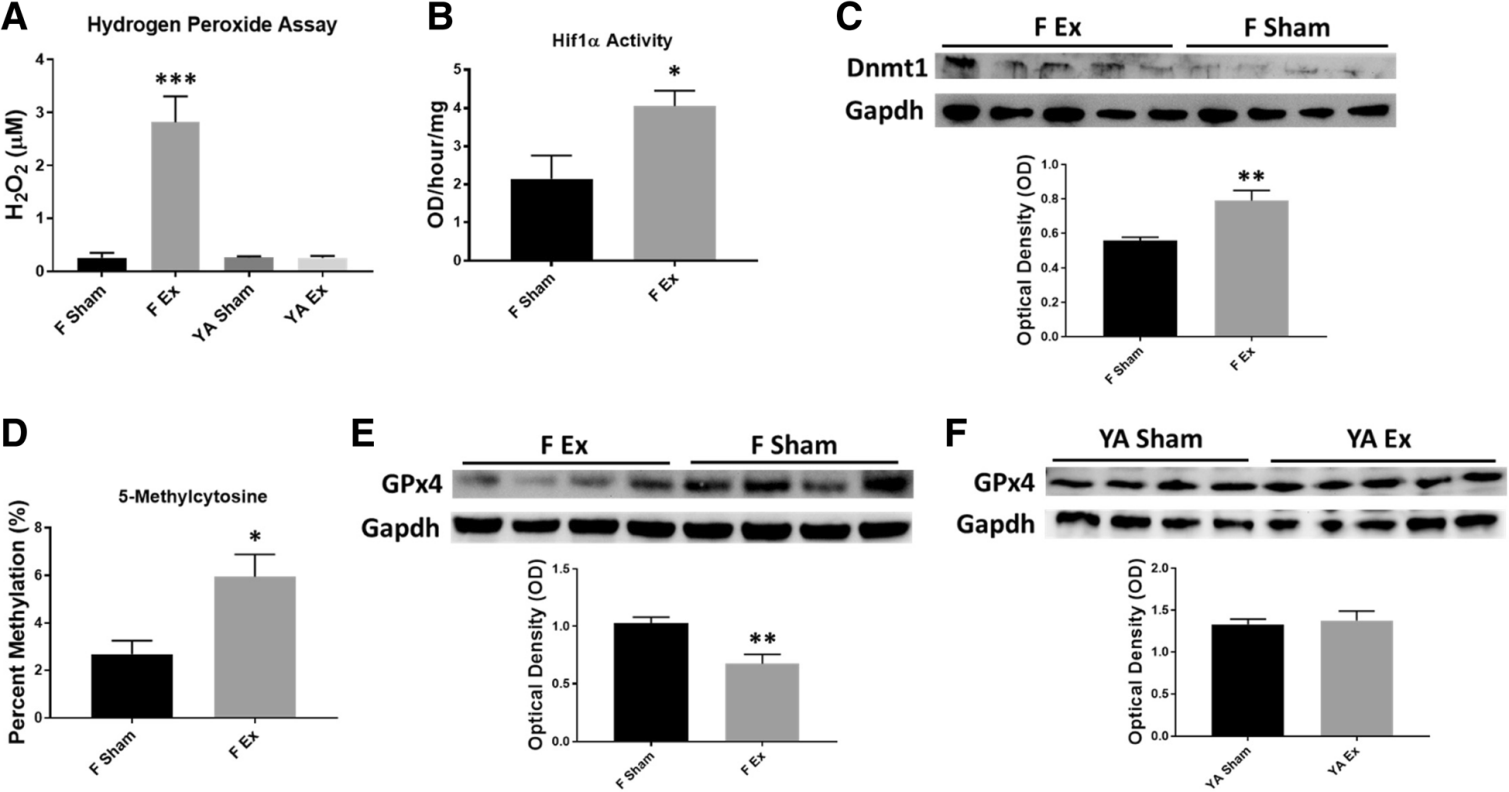
Mechanisms governing cardiac and mitochondrial dysfunction following maternal nano-TiO_2_ inhalation exposure. **a** Hydrogen peroxide (H_2_O_2_) concentration in fetal (*n* = 6 Sham, *n* = 5 Ex) and young adult (*n* = 7 Sham, *n* = 5 Ex) animals, normalized to protein content. **b** Hif1α activity was measured in fetal (*n* = 4 Sham, *n* = 5 Ex) heart and normalized to protein content. **c** Dnmt1 protein expression was assessed in fetal (*n* = 4 Sham, *n* = 5 Ex) heart and normalized using anti-Gapdh primary antibody. **d** Global 5-methylcytosine (5-mC) DNA methylation levels were evaluated in fetal (*n* = 6 Sham, *n* = 5 Ex) hearts, normalized to DNA concentration. **e** GPx4 levels were assessed in fetal (*n* = 4 Sham, *n* = 4 Ex) and (**f**) young adult (*n* = 4 Sham, *n* = 5 Ex) animals and normalized using anti-Gapdh primary antibody. Sham = control filtered air exposed, Ex = nano-TiO_2_ exposed, Maternal (M) = 12-week old pregnant dams, Fetal (F) = GD (15), Young Adult (YA) = 11 weeks, Hif1α = Hypoxia-inducible factor 1-alpha, Dnmt1 = DNA (cytosine-5)-methyltransferase 1, GPx4 = Glutathione peroxidase 4. All data are presented as the mean ± standard error of the mean (SEM). * = *P* ≤ 0.05, ** = *P* ≤ 0.01, *** = *P* ≤ 0.001 for Ex vs. Sham

H_2_O_2_ production was assessed in maternal, fetal, and young adult hearts, placenta, as well as in fetal and young adult liver and lung. There was a significant increase in H_2_O_2_ content in the fetal hearts of pups whose dams were exposed to nano-TiO_2_ (Fig. 4a), with a decrease in H_2_O_2_ content in the liver (Additional file 2: Figure S2A). There were no significant changes in H_2_O_2_ content in the young adult offspring heart (Fig. 4a) or other young adult tissues (Additional file 2: Figure S2A). Hif1α activity was measured to assess how H_2_O_2_ may influence the transcription factor activity and binding. Hif1α activity was significantly increased in the hearts of fetal offspring whose dams were exposed to nano-TiO_2_ (Fig. 4b) as well as in the maternal heart (Additional file 2: Figure S2B). Intriguingly, there was a decrease in activity in the fetal lung and liver tissues following nano-TiO_2_ inhalation exposure (Additional file 2: Figure S2B).

Because Hif1α is a regulator of Dnmt1 expression, we determined whether H_2_O_2_, induced through nano-TiO_2_ inhalation exposure, was concomitant with increased transcription of Dnmt1 and, ultimately, DNA methylation patterns. Dnmt1 protein expression in the fetal heart reveled a significant increase in pups whose dams were exposed to nano-TiO_2_ (Fig. 4c). Although there was an increase in Dnmt1 protein expression following exposure, Dnmt activity was not significantly altered (Additional file 2: Figure S2C). Additionally, we found that Dnmt3b expression was not changed, following maternal inhalation exposure to ENM in fetal offspring (Additional file 2: Figure S2D). Global DNA methylation levels displayed a significant increase in the hearts of the pups whose dams were exposed to nano-TiO_2_ (Fig. 4d). While we observed an increase in 5-mC DNA methylation in fetal pups whose dams were exposed to nano-TiO_2_, development into adulthood revealed an inverse relationship, with a significant decrease in global DNA methylation (Additional file 2: Figure S2E). Augmented 5-mC DNA methylation levels at the fetal stage indicate that maternal nano-TiO_2_ exposure during gestation potentially results in the repression of vital genes, which could lead to detrimental dysfunction. Furthermore, cardiac DNA methylation at the fetal and young adult stages reveal an aberrant DNA methylation pattern, which may be attributed to the Hif1α/Dnmt1 regulatory axis.

### GPx4 expression and mitochondrial structure

We next determined whether enhanced H_2_O_2_ levels are associated with the repression of GPx4 protein expression. A significant diminution of GPx4 protein expression was revealed in the fetal hearts of pups whose dams were exposed to nano-TiO_2_ during gestation (Fig. 4e), while GPx4 protein levels were unchanged in the young adult offspring (Fig. 4f).

**Fig. 5:**
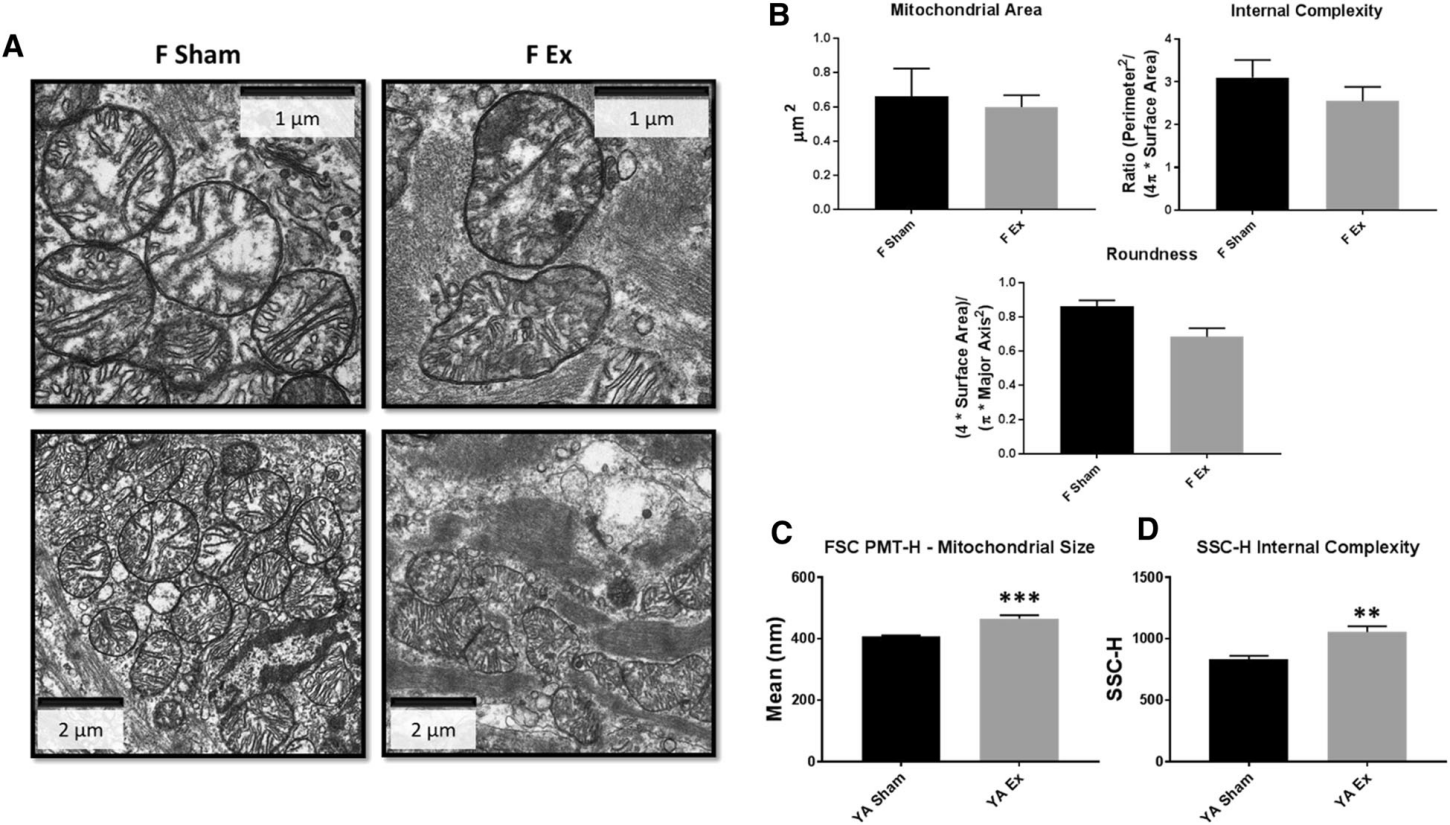
Mitochondrial ultrastructure following maternal nano-TiO_2_ inhalation exposure. **a** Transmission electron micrographs (TEM) of fetal cardiac tissue following Sham and nano-TiO_2_ maternal inhalation exposure. **b** Mitochondrial area, internal complexity (form factor), and roundness (0–1, where 1 = perfect spheroid) were assessed through TEM (*n* = 2 Sham, *n* = 2 Ex). **c** Mitochondrial size and (**d**) internal complexity were determined in young adult (*n* = 7 Sham, *n* = 5 Ex) animals through FSC and SSC gating, staining mitochondria with MitoTracker™ Deep Red FM/633. Sham = control filtered air exposed, Ex = nano-TiO_2_ exposed, Maternal (M) = 12-week old pregnant dams, Fetal (F) = GD (15), Young Adult (YA) = 11 weeks, FSC = Forward Scatter, SSC = Side Scatter. All data are presented as the mean ± standard error of the mean (SEM). * = *P* ≤ 0.05, ** = *P* ≤ 0.01, *** = *P* ≤ 0.001 for Ex vs. Sham

To determine if exposure to nano-TiO_2_ affected fetal mitochondrial ultrastructure, TEM was implemented to assess mitochondrial size and complexity (Fig. 5a). Using a semi-quantitative approach, mitochondrial area and internal complexity were shown to be similar between the Sham and nano-TiO_2_ groups, while mitochondrial roundness revealed a decrease in the hearts of the maternal nano-TiO_2_ exposed fetal offspring (Fig. 5b). To gain insight into the impact of gestational nano-TiO_2_ exposure on mitochondrial ultrastructure at the young adult stage, mitochondrial size (Fig. 5c) and complexity (Fig. 5d) were evaluated through flow cytometry. FSC (size) and SSC (internal complexity) were significantly increased in young adult offspring whose dams were exposed to nano-TiO_2_ during gestation. These data are suggestive of an alteration to mitochondrial ultrastructure as a result of gestational nano-TiO_2_ exposure; the altered shape of fetal mitochondria could potentially influence the chronic changes to mitochondrial size and internal complexity in the young adults, precipitating sustained bioenergetic and cardiac dysfunction into adulthood.

## Discussion

The use of nanotechnology continues to increase in both the variety of applicable fields, as well as the prevalence within those fields. Nano-TiO_2_’s high refractive index offers high opacity and resistance to corrosion making it beneficial in the industries of paints, inks, papers, and plastics [10]. Unfortunately, the repercussions of the rampant use of ENMs remain unclear, along with the mechanisms and solutions to the potential negative consequences. In order to determine how the current exposure paradigm, which achieved a lung burden of 95.10 μg over 6 days, reflects ENM occupational exposure in humans in a manufacturing setting, alveolar surface area was used as previously described [11, 13, 51, 52]. The mouse alveolar surface area is 0.05 m^2^ [52]. Therefore, the mouse lung burden of 95.10 μg would result in 1902 μg/m^2^. Since the human alveolar surface area is 102 m^2^, the human lung burden with this exposure paradigm would be 194.0 mg. Furthermore, the number of working days it would take to achieve this lung burden in humans was calculated:

$$ {\displaystyle \begin{array}{l} nano- TiO2\; aerosol\kern0.17em concentration\cdot \mathit{\min} ute\; ventilation\\ {}\kern1.68em \cdot \mathit{\exp} osure\kern0.17em duration\cdot deposition\kern0.17em fraction,\end{array}} $$

with the following values:

$$ {\displaystyle \begin{array}{l}194.0\; mg= nano- TiO2\; aerosol\kern0.17em concentration\\ {}\kern0.36em \cdot 7600\; ml/\mathit{\min}\cdot \left(8 hrs/ day\cdot 60\;\mathit{\min}/ hr\right)\cdot 14\%,\end{array}} $$

therefore:

$$ {\displaystyle \begin{array}{lll}194.0\kern0.3em mg& =& nano- TiO2\kern0.28em aerosol\kern0.34em concentration\\ {}& & \kern3.079998em \cdot 0.51\kern0.3em {m}^3/ day\\ {}76\kern0.3em days& =& 194.0\kern0.28em mg/\left(\left(5\kern0.28em mg/{m}^3\right)\cdot 0.51\kern0.28em {m}^3\right).\end{array}} $$

The National Institute for Occupational Safety and Health (NIOSH) Recommended Exposure Limit for nano-TiO_2_ aerosol concentration is 0.3 mg/m^3^, while the Permissible Exposure Limit set by the Occupational Safety and Health Administration is 5 mg/ m^3^ [13]. Consequently, it would require 76 days for a human to achieve analogous lung burdens with the exposure paradigm used in this study. Thus, the findings are of practical relevance for those exposed in an occupational context. Importantly, our results demonstrate that H_2_O_2_-induced stress from maternal nano-TiO_2_ inhalation exposure impacts global DNA methylation remodeling, and is associated with sustained mitochondrial bioenergetic and cardiac contractile dysfunction.

Functioning mitochondria are necessary for providing the immense energy requirement that drives cardiac contraction and relaxation. Thus, mitochondrial dysfunction has been widely implicated as a precipitator of cardiac contractile dysfunction. ROS, such as H_2_O_2_, are a by-product of oxidative phosphorylation, and as a result, the mitochondrion is an initial site of generation and damage. Pathological conditions, such as those presented by ENM exposure, have been associated with enhanced ROS production and mitochondrial dysfunction in cardiac tissue that may precede contractile dysfunction [14]. H_2_O_2_ accumulation in the heart following maternal inhalation exposure to ENMs was significantly associated with negative consequences in the growing fetus, but the direct link between maternal exposure and increased progeny cardiac H_2_O_2_ is unknown. One theory suggests that changes in the placental environment, through changes in circulatory flow or inflammation, can trigger tissue specific alterations in the progeny [53].

In the current study we have shown that umbilical blood flow, through a reduction in end-diastolic velocity, was significantly altered (Fig. 2e). Following an ENM exposure paradigm similar to the paradigm used for the current study, an increase in placental vascular resistance was reported, substantiating the antagonistic effects of nano-TiO_2_ exposure during gestation [19]. Valentino et al. further validate placental circulatory remodeling through a significant decline in placental flow in rabbits, which was observed following gestational exposure to diesel exhaust [54]. Additionally, they report decreased vascularization of the placental bed contributing to the effect. Along with diminished maternal-to-fetal blood flow following inhaled gases and particulates, an inflammatory response promoted through the vasculature could also be a contributing factor in untoward cardiovascular events in the growing progeny. Interleukin 1 beta (IL-1β), IL-6, and monocyte chemoattractant protein-1 (MCP-1) were shown to be significantly elevated in maternal serum following inhalation exposure to ultrafine particulate matter [55]. While the direct link between cardiac ROS production/accumulation and alterations to the placental environment are currently unknown, a strong link between placental mitochondrial health (Additional file 2: Figures S1C-E) and fetal cardiovascular outcomes is undoubtedly present. Following maternal ENM inhalation exposure, direct interaction of the ENM with the fetus, maternal immune responses, or other responses involving alterations in blood supply or content could all contribute to increased ROS in the cardiac tissue of the growing fetus. This increased fetal ROS, which is returned to baseline in the young adult, likely contributes to sustained cellular consequences, such as abnormal mitochondrial bioenergetics and ultrastructure which have been observed following ENM exposure, as well as disruption to the epigenome [13, 24].

Under normal conditions, oxygen-dependent prolyl hydroxylases target Hif1α for proteasomal degradation; however, during hypoxic conditions, such as when ROS levels are elevated, prolyl hydroxylase activity is limited by the lack of oxygen and therefore results in the stabilization of Hif1α permitting dimerization with Hif1β, which is constitutively expressed [56–59]. This promotes the formation of an active HIF transcription factor complex [60]. The Dnmt1 and Dnmt3b promoter regions contain the hypoxia response element (HRE), a consensus sequence for a Hif1α binding site [61]. We found a significant increase in Dnmt1 protein expression, but not activity, further substantiating our hypothesis that in a high ROS environment, elevated Hif1α activity leads to hypermethylation by causing an increase in Dnmt1 protein expression through promoter binding. DNA hypermethylation can cause enhanced profibrotic gene expression and the hypermethylation of CpG islands of vital genes, causing loss of gene expression [61]. This could in turn propagate mitochondrial dysfunction and increased size associated with ENM exposure and increased ROS.

While the direct effects of ROS, such as disruption of protein structure and function, pose a significant threat to cellular health, indirect effects, such as ROS-mediated epigenetic remodeling, can have equal, if not more pronounced, ramifications. In melanoma cells, detachment from the plating surface revealed an increase in ROS, including H_2_O_2_, as well as an increase in global DNA methylation and Dnmt1 expression [62]. When introducing an antioxidant, global DNA hypermethylation and Dnmt1 increased expression was shown to be prevented. Outside of global methylation, ROS can induce site-specific methylation profiles. H_2_O_2_ has been shown to increase DNMT1 expression, resulting in hypermethylation and decreased expression of runt domain transcription factor 3 (RUNX3) [63]. Additionally, increased H_2_O_2_ has been linked to DNA hypermethylation and silencing of miRNAs, which regulate the expression of multiple genes and gene pathways in the cell [64]. ROS have been linked to epigenetic modification of the promoter region of other glutathione peroxide family members (GPX3) [65].

**Fig. 6:**
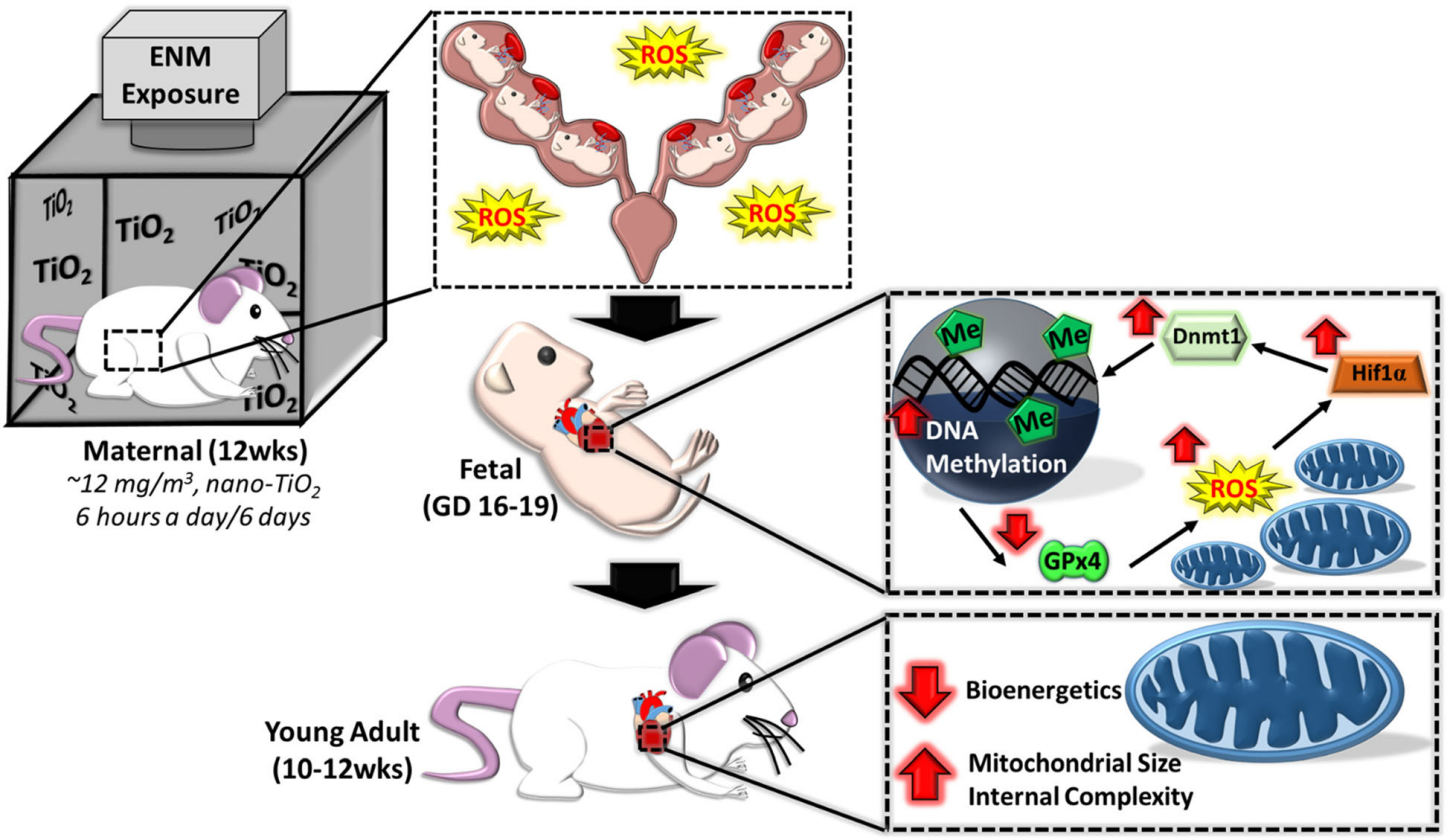
Illustration of molecular pathways altered during maternal nano-TiO_2_ inhalation exposure and physiological ramifications. Following maternal exposure, increased ROS in fetal cardiac tissue and decreased mitochondrial ROS scavenging through GPx4 perpetuates a positive feedback-loop where increased Hif1α activity acts to transcriptionally activate Dnmt1 and ultimately increase global 5-methylcytosine levels. While ROS returns to control levels in the young adult animals, fetal insult negatively influences mitochondrial and cardiac function in maternal nano-TiO_2_ inhalation exposed progeny into adulthood. Maternal (M) = 12-week-old pregnant dams, Fetal (F) = GD (15), Young Adult (YA) = 11 weeks, ROS = reactive oxygen species, GPx4 = Glutathione peroxidase 4, Hif1α = Hypoxia-inducible factor 1-alpha, Dnmt1 = DNA (cytosine-5)-methyltransferase 1, Me = 5-methylcytosine

The schema provided in Fig. 6 encompasses the overall mechanism hypothesized based on the data in the current study. At the fetal stage, cardiac function is negatively impacted through increased H_2_O_2_ levels, which can have negative consequences on mitochondrial function and bioenergetics. The increased H_2_O_2_ can also lead to an activation of Hif1α activity, which is then able to transcriptionally activate the promoter region of a variety of genes, including Dnmt1 and other epigenetic machinery. Augmenting Dnmt1 expression would lead to a global or site-specific increase in methylation, and in turn repress pivotal genes such as GPx4. By decreasing antioxidant defenses (GPx4), a futile cycle would be propagated through a decreased ability to scavenge ROS such as H_2_O_2_, resulting in a further increase in ROS. Cardiac and mitochondrial dysfunction are therefore sustained into adulthood due to epigenomic remodeling that occurred at the fetal stage.

The role of epigenetic remodeling has been investigated in terms of the intrauterine environment and external stressors may have negative implications on the growing fetus [9, 66]. The offspring of mothers who had diabetes mellitus or were obese during gestation showed increased incidence of epigenetic changes and an increased risk of type 2 diabetes mellitus and other metabolic disorders that can lead to cardiovascular dysfunction [67]. Alterations in the epigenome of young adult offspring were also shown as a potential result of ENM exposure in pregnant rats, which could enhance susceptibility to future insult [13]. The demonstration of such changes in a mouse model, as detailed in this study, opens up the possibility of examining the fetal and young adult offspring epigenome using genetic manipulations [14, 24].

This study suggests potential mechanisms such as ROS, that can contribute to epigenomic remodeling of the fetus, in utero, but the study is limited in unveiling larger pathways associated with increased genome methylation. We provide a mechanism whereby ROS scavenging is decreased, leading to the accumulation of ROS and ultimately methylation of the genome, but it is likely that this pathway is more dynamic with other epigenetic machinery and transcription factors that are altered. Future experimentation into the specific genes and regulatory pathways that are hypermethylated may provide better insight into the mechanisms contributing to the sustained mitochondrial and cardiac dysfunction observed.

## Conclusions

This study highlights disruptions in cardiac and mitochondrial function in offspring of nano-TiO_2_ exposed mice during gestation. These dysfunctions are sustained into adulthood and are most likely due to epigenetic reprogramming, mediated through enhanced H_2_O_2_ which occurs during gestation in the growing fetus. Increased methylation and decreased GPx4 levels suggest repression of important antioxidant proteins, thus perpetuating the inability to control elevated ROS levels and leading to mitochondrial and cardiac dysfunction.

## Additional files

Additional file 1: Supplemental tables to the primary manuscript including, Pulse-Wave Doppler-Flow for umbilical and uterine flow, as well as diastolic and systolic cardiac stress- strain in the short and long axes for maternal, fetal, and young adult animals. These additional parameters are provided in order to ensure a thorough assessment of cardiac contractile function and for support of our conclusions. (DOCX 27 kb)

Additional file 2: Supplemental figures to the primary manuscript including, mitochondrial bioenergetics of other tissues and the assessment of ROS mediated pathways in other tissues and organ systems in maternal, fetal, and young adult animals. These additional parameters are provided in order to ensure a thorough assessment and further insight into the ROS-related consequences of maternal nano-TiO_2_ inhalation exposure during gestation. (DOCX 267 kb)

## Data Availability

All data generated or analyzed during this study are included in this published article [and its Additional files].
