# Supplementary material for: ROS promote epigenetic remodeling and cardiac dysfunction in offspring following maternal engineered nanomaterial (ENM) exposure
Source: Part Fibre Toxicol. 2019 Jun 18;16:24. doi: 10.1186/s12989-019-0310-8 (PMC6582485; doi:10.1186/s12989-019-0310-8)
Supplement: Supplementary file 1 — Supplemental tables to the primary manuscript including, Pulse-Wave Doppler-Flow for umbilical and uterine flow, as well as diastolic and systolic cardiac stress- strain in the short and long axes for maternal, fetal, and young adult animals. These additional parameters are provided in order to ensure a thorough assessment of cardiac contractile function and for support of our conclusions. (DOCX 27 kb) [file 12989_2019_310_MOESM1_ESM.docx]

**ROS promote epigenetic remodeling and cardiac dysfunction in offspring following maternal engineered nanomaterial (ENM) exposure**

Amina Kunovac^1,2,4^, Quincy A. Hathaway^1,2,4^, Mark V. Pinti^3^, William T. Goldsmith^4,5^, Andrya J. Durr ^1,2^, Garrett K. Fink,^1^, Timothy R. Nurkiewicz^4,5^, and John M. Hollander^1,2^

**^1^**Division of Exercise Physiology, West Virginia University School of Medicine, Morgantown, WV, USA. **^2^**Mitochondria, Metabolism & Bioenergetics Working Group, West Virginia University School of Medicine, Morgantown, WV, USA. **^3^**West Virginia University School of Pharmacy, Morgantown, WV, USA. **^4^**Center for Inhalation Toxicology (iTOX), West Virginia University School of Medicine, Morgantown, WV, USA. **^5^**Department of Physiology, Pharmacology, Morgantown, WV, USA.

Amina Kunovac; [ak0086@mix.wvu.edu](mailto:ak0086@mix.wvu.edu), Quincy A. Hathaway; [qahathaway@mix.wvu.edu](mailto:qahathaway@mix.wvu.edu), Mark V. Pinti; [mpinti@mix.wvu.edu](mailto:mpinti@mix.wvu.edu), William T. Goldsmith; [wgoldsmi@hsc.wvu.edu](mailto:wgoldsmi@hsc.wvu.edu), Andrya J. Durr; [ajdurr@mix.wvu.edu](mailto:ajdurr@mix.wvu.edu), Garrett K. Fink; [gkf0001@mix.wvu.edu](mailto:gkf0001@mix.wvu.edu), Timothy R. Nurkiewicz; [tnurkiewicz@hsc.wvu.edu](mailto:tnurkiewicz@hsc.wvu.edu), John M. Hollander; [jhollander@hsc.wvu.edu](mailto:jhollander@hsc.wvu.edu)

Corresponding Author:

John M. Hollander, Ph.D., F.A.H.A.

Division of Exercise Physiology

West Virginia University School of Medicine

PO Box 9227

1 Medical Center Drive

Morgantown, WV 26506

Tel: 1-(304) 293-3683

Fax: 1-(304) 293-7105

Email: [jhollander@hsc.wvu.edu](mailto:jhollander@hsc.wvu.edu)

**Supplemental Table Legends**

**Additional file 1: Table S1.** **Pulse Wave Doppler-Flow echocardiography for umbilical and uterine flow following maternal nano-TiO_2_ exposure.** Maternal (n = 12 Sham, n = 8 Ex). Sham = control filtered air exposed, Ex = nano-TiO_2_ exposed, Maternal (M) = 12-week old pregnant dams. All data are presented as the mean ± standard error of the mean (SEM). ** = *P* ≤ 0.01 for Ex vs. Sham.

**Additional file 1: Table S2. Diastole cardiac stress-strain in the short axis.** B-Mode images were used to examine radial and circumferential stress-strain parameters in the short axis during diastole in maternal (n = 11 Sham, n = 15 Ex), fetal (n = 9 Sham, n = 9 Ex), and young adult (n = 7 Sham, n = 5 Ex) animals. Sham = control filtered air exposed, Ex = nano-TiO_2_ exposed, Maternal (M) = 12-weekold pregnant dams, Fetal (F) = GD (15), Young Adult (YA) = 11 weeks. All data are presented as the mean ± standard error of the mean (SEM). * = *P* ≤ 0.05, ** = *P* ≤ 0.01 for Ex vs. Sham.

**Additional file 1: Table S3.** **Systolic cardiac stress-strain in the short axis.** B-Mode images were used to examine radial and circumferential stress-strain parameters in the short axis during systole in maternal (n = 11 Sham, n = 15 Ex), fetal (n = 9 Sham, n = 9 Ex), and young adult (n = 7 Sham, n = 5 Ex) animals. Sham = control filtered air exposed, Ex = nano-TiO_2_ exposed, Maternal (M) = 12-week old pregnant dams, Fetal (F) = GD (15), Young Adult (YA) = 11 weeks. All data are presented as the mean ± standard error of the mean (SEM). * = *P* ≤ 0.05, ** = *P* ≤ 0.01 for Ex vs. Sham.

**Additional file 1: Table S4.** **Diastolic cardiac stress-strain in the long axis.** B-Mode images were used to examine longitudinal and circumferential stress-strain parameters in the long axis during diastole in maternal (n = 11 Sham, n = 15 Ex), fetal (n = 9 Sham, n = 9 Ex), and young adult (n = 7 Sham, n = 5 Ex) animals. Sham = control filtered air exposed, Ex = nano-TiO_2_ exposed, Maternal (M) = 12-week old pregnant dams, Fetal (F) = GD (15), Young Adult (YA) = 11 weeks. All data are presented as the mean ± standard error of the mean (SEM). * = *P* ≤ 0.05 for Ex vs. Sham.

**Additional file 1: Table S5.** **Systolic cardiac stress-strain in the long axis.** B-Mode images were used to examine longitudinal and circumferential stress-strain parameters in the long axis during systole in maternal (n = 11 Sham, n = 15 Ex), fetal (n = 9 Sham, n = 9 Ex), and young adult (n = 7 Sham, n = 5 Ex) animals. Sham = control filtered air exposed, Ex = nano-TiO_2_ exposed, Maternal (M) = 12-week old pregnant dams, Fetal (F) = GD (15), Young Adult (YA) = 11 weeks. All data are presented as the mean ± standard error of the mean (SEM). * = *P* ≤ 0.05, ** = *P* ≤ 0.01 for Ex vs. Sham.

**Additional file 1: Table S1. Pulse-Wave Doppler Maternal Flow**

| **Parameters** | **Units** | **M Sham Uterine** | **M Ex Uterine** | **M Sham Umbilical** | **M Ex Umbilical** |
| --- | --- | --- | --- | --- | --- |
| **End-Diastolic Velocity** | mm/s | 46.66 ± 4.203 | 45.31 ± 6.686 | 9.864 ± 1.287 | **3.994 ± 0.792**** |
| **Peak-Systolic Velocity** | mm/s | 129.3 ± 14.26 | 112.6 ± 11.57 | 84.04 ±10.20 | 78.03 ± 7.367 |
| **Velocity Time Integral** | (VTI) mm | 8.252 ± 0.786 | 8.296 ± 1.192 | 9.433 ± 0.907 | 8.012 ± 0.924 |
| **Velocity Time Integral** | (Mean Vel) mm/s | 81.54 ± 8.967 | 74.12 ± 8.954 | 36.83 ± 4.997 | 33.81 ± 3.237 |
| **Velocity Time Integral** | (Mean Grad) mmHg | 0.025 ± 0.004 | 0.024 ± 0.005 | 0.007 ± 0.002 | 0.005 ± 0.001 |
| **Velocity Time Integral** | (Peak Vel) mm/s | 124.8 ± 12.46 | 118.79 ± 12.88 | 84.43 ± 10.15 | 78.14 ± 7.381 |
| **Velocity Time Integral** | (Peak Grad) mmHg | 0.077 ± 0.017 | 0.061 ± 0.012 | 0.033 ± 0.008 | 0.026 ± 0.004 |

**Additional file 1: Table S2. Stress-Strain Analysis – Short Axis – Diastole**

| **Parameters** | **Units** | **M Sham** | **M Ex** | **F Sham** | **F Ex** | **YA Sham** | **YA Ex** |
| --- | --- | --- | --- | --- | --- | --- | --- |
| **Heart Rate** | beats/min | 670.1 ± 9.4 | 684.2 ± 9.1 | 123.6 ± 8.186 | 123.9 ± 9.679 | 632.1 ± 24.78 | 689.3 ± 8.156 |
| **Radial Velocity (Endo)** | Pk cm/s | -3.913 ± 0.13 | -3.336 ± 0.324 | -0.227 ± 0.033 | -0.163 ± 0.011 | -2.210 ± 0.262 | -2.481 ± 0.366 |
| **Radial Displacement (Endo)** | Pk mm | -0.004 ± 0.001 | -0.008 ± 0.002 | -0.005 ± 0.005 | -0.011 ± 0.004 | -0.0002 ± 0.0001 | **-0.020 ± 0.009*** |
| **Radial Strain (Endo)** | Pk % | -4.192 ± 1.288 | -2.497 ± 0.536 | -29.80 ± 7.744 | -13.28 ± 3.711 | -2.687 ± 1.264 | -8.834 ± 4.281 |
| **Radial Strain Rate (Endo)** | Pk 1/s | -29.26 ± 4.182 | -22.28 ± 1.672 | -54.07 ± 4.292 | -60.89 ± 4.576 | -16.58 ± 2.631 | -30.13 ± 10.87 |
| **Circumferential Velocity (Endo)** | Pk deg/s | -698.1 ± 94.69 | -811.1 ± 155.1 | -444.17 ± 83.60 | -398.8 ± 51.49 | -608.8 ± 67.48 | -473.5 ± 1215.5 |
| **Circumferential Displacement (Endo)** | Pk deg | -2.101 ± 0.603 | -3.930 ± 1.974 | -6.367 ± 1.440 | **-16.62 ± 3.183**** | -6.108 ± 2.036 | -6.623 ± 4.394 |
| **Circumferential Strain (Endo)** | Pk % | 5.644 ± 1.792 | 2.076 ± 0.646 | 9.881 ± 4.341 | 7.888 ± 2.411 | 22.89 ± 21.61 | 15.58 ± 8.565 |
| **Circumferential Strain Rate (Endo)** | Pk 1/s | 47.67 ± 2.911 | 51.29 ± 3.699 | 12.660 ± 1.975 | 10.58 ± 1.394 | 38.43 ± 2.817 | 47.02 ± 11.50 |

**Additional file 1: Table S3. Stress-Strain Analysis – Short Axis – Systole**

| **Parameters** | **Units** | **M Sham** | **M Ex** | **F Sham** | **F Ex** | **YA Sham** | **YA Ex** |
| --- | --- | --- | --- | --- | --- | --- | --- |
| **Heart Rate** | beats/min | 670.1 ± 9.4 | 684.2 ± 9.1 | 123.6 ± 8.186 | 123.9 ± 9.679 | 632.1 ± 24.78 | 689.3 ± 8.156 |
| **Radial Velocity (Endo)** | Pk cm/s | 2.951 ± 0.133 | 2.854 ± 0.170 | 0.194 ± 0.035 | 0.173 ± 0.014 | 2.404 ± 0.258 | 2.754 ± 0.317 |
| **Radial Displacement (Endo)** | Pk mm | 0.769 ± 0.022 | 0.700 ± 0.049 | 0.143 ± 0.012 | 0.128 ± 0.009 | 0.549 ± 0.060 | 0.629 ± 0.057 |
| **Radial Strain (Endo)** | Pk % | 39.26 ± 3.328 | **52.37 ± 4.428*** | 10.76 ± 0.960 | 11.82 ± 2.382 | 35.49 ± 2.510 | 39.28 ± 19.79 |
| **Radial Strain Rate (Endo)** | Pk 1/s | 19.35 ± 2.023 | 19.47 ± 1.664 | 54.07 ± 9.664 | **8.464 ± 1.889**** | 27.10 ± 6.571 | 25.22 ± 8.153 |
| **Circumferential Velocity (Endo)** | Pk deg/s | 695.5 ± 104.4 | 810.9 ± 113.9 | 404.25 ± 68.07 | 356.7 ± 37.99 | 724.9 ± 143.5 | 440.85 ± 46.91 |
| **Circumferential Displacement (Endo)** | Pk deg | 6.384 ± 0.652 | 4.883 ± 0.817 | 11.62 ± 2.265 | **2.954 ± 0.724**** | 3.190 ± 0.899 | 4.512 ± 1.424 |
| **Circumferential Strain (Endo)** | Pk % | -38.54 ± 4.448 | **-54.51 ± 2.811*** | -27.86 ± 2.405 | -25.12 ± 3.225 | -39.90 ± 7.110 | -39.94 ± 8.000 |
| **Circumferential Strain Rate (Endo)** | Pk 1/s | -44.57 ± 4.357 | -46.94 ± 4.056 | -12.63 ± 1.676 | -8.919 ± 0.432 | -45.53 ± 4.426 | -45.22 ± 12.34 |

**Additional file 1: Table S4. Stress-Strain Analysis – Long Axis – Diastole**

| **Parameters** | **Units** | **M Sham** | **M Ex** | **YA Sham** | **YA Ex** |
| --- | --- | --- | --- | --- | --- |
| **Heart Rate** | beats/min | 681.3 ± 11.66 | 708.0 ± 11.75 | 581.9 ± 54.00 | 679.1 ± 4.952 |
| **Radial Velocity (Endo)** | Pk cm/s | -2.640 ± 0.146 | **-2.152 ± 0.128*** | -1.691 ± 0.043 | -1.736 ± 0.121 |
| **Radial Displacement (Endo)** | Pk mm | -0.006 ± 0.002 | -0.002 ± 0.001 | -0.003 ± 0.001 | -0.007 ± 0.003 |
| **Radial Strain (Endo)** | Pk % | -1.047 ± 0.234 | -0.782 ± 0.213 | -1.413 ± 0.280 | -0.725 ± 0.282 |
| **Radial Strain Rate (Endo)** | Pk 1/s | -14.25 ± 0.980 | **-10.94 ± 0.933*** | -9.768 ± 0.949 | -9.205 ± 1.083 |
| **Longitudinal Velocity (Endo)** | Pk deg/s | -1.579 ± 0.246 | -1.690 ± 0.152 | -1.105 ± 0.119 | -0.873 ± 0.169 |
| **Longitudinal Displacement (Endo)** | Pk deg | -0.021 ± 0.006 | -0.034 ± 0.010 | -0.140 ± 0.034 | **-0.036 ± 0.016*** |
| **Longitudinal Strain (Endo)** | Pk % | 4.654 ± 1.262 | **0.933 ± 0.352*** | 2.209 ± 0.737 | 1.841 ± 0.528 |
| **Longitudinal Strain Rate (Endo)** | Pk 1/s | 9.834 ± 1.207 | 11.89 ± 1.223 | 9.890 ± 1.586 | 9.867 ± 2.347 |

**Additional file 1: Table S5. Stress-Strain Analysis – Long Axis – Systole**

| **Parameters** | **Units** | **M Sham** | **M Ex** | **YA Sham** | **YA Ex** |
| --- | --- | --- | --- | --- | --- |
| **Heart Rate** | beats/min | 681.3 ± 11.66 | 708.0 ± 11.75 | 581.9 ± 54.00 | 679.1 ± 4.952 |
| **Radial Velocity (Endo)** | Pk cm/s | 2.054 ± 0.091 | **1.787 ± 0.060*** | 2.003 ± 0.142 | 1.954 ± 0.070 |
| **Radial Displacement (Endo)** | Pk mm | 0.574 ± 0.023 | **0.469 ± 0.022**** | 0.468 ± 0.037 | 0.479 ± 0.038 |
| **Radial Strain (Endo)** | Pk % | 33.33 ± 2.797 | 27.53 ± 2.388 | 27.39 ± 3.189 | 27. 33 ±0.595 |
| **Radial Strain Rate (Endo)** | Pk 1/s | 12.07 ± 1.203 | 10.94 ± 1.357 | 9.365 ± 0.809 | 10.49 ± 0.621 |
| **Longitudinal Velocity (Endo)** | Pk deg/s | 1.245 ± 0.131 | 1.466 ± 0.092 | 0.997 ± 0.215 | 0.923 ± 0.248 |
| **Longitudinal Displacement (Endo)** | Pk deg | 0.237 ± 0.040 | 0.247 ± 0.035 | 0.065 ± 0.011 | 0.100 ± 0.022 |
| **Longitudinal Strain (Endo)** | Pk % | -7.070 ± 1.597 | -10.40 ± 1.741 | -12.15 ± 2.510 | -16.87 ± 2.052 |
| **Longitudinal Strain Rate (Endo)** | Pk 1/s | -7.309 ± 2.845 | **-11.80 ± 1.789*** | -10.40 ± 1.282 | -8.526 ± 1.306 |
