# Supplementary material for: ROS promote epigenetic remodeling and cardiac dysfunction in offspring following maternal engineered nanomaterial (ENM) exposure
Source: Part Fibre Toxicol. 2019 Jun 18;16:24. doi: 10.1186/s12989-019-0310-8 (PMC6582485; doi:10.1186/s12989-019-0310-8)

**ROS promote epigenetic remodeling and cardiac dysfunction in offspring following maternal engineered nanomaterial (ENM) exposure**

Amina Kunovac^1,2,4^, Quincy A. Hathaway^1,2,4^, Mark V. Pinti^3^, William T. Goldsmith^4,5^, Andrya J. Durr ^1,2^, Garrett K. Fink,^1^, Timothy R. Nurkiewicz^4,5^, and John M. Hollander^1,2^

**^1^**Division of Exercise Physiology, West Virginia University School of Medicine, Morgantown, WV, USA. **^2^**Mitochondria, Metabolism & Bioenergetics Working Group, West Virginia University School of Medicine, Morgantown, WV, USA. **^3^**West Virginia University School of Pharmacy, Morgantown, WV, USA. **^4^**Center for Inhalation Toxicology (iTOX), West Virginia University School of Medicine, Morgantown, WV, USA. **^5^**Department of Physiology, Pharmacology, Morgantown, WV, USA.

Amina Kunovac; [ak0086@mix.wvu.edu](mailto:ak0086@mix.wvu.edu), Quincy A. Hathaway; [qahathaway@mix.wvu.edu](mailto:qahathaway@mix.wvu.edu), Mark V. Pinti; [mpinti@mix.wvu.edu](mailto:mpinti@mix.wvu.edu), William T. Goldsmith; [wgoldsmi@hsc.wvu.edu](mailto:wgoldsmi@hsc.wvu.edu), Andrya J. Durr; [ajdurr@mix.wvu.edu](mailto:ajdurr@mix.wvu.edu), Garrett K. Fink; [gkf0001@mix.wvu.edu](mailto:gkf0001@mix.wvu.edu), Timothy R. Nurkiewicz; [tnurkiewicz@hsc.wvu.edu](mailto:tnurkiewicz@hsc.wvu.edu), John M. Hollander; [jhollander@hsc.wvu.edu](mailto:jhollander@hsc.wvu.edu)

Corresponding Author:

John M. Hollander, Ph.D., F.A.H.A.

Division of Exercise Physiology

West Virginia University School of Medicine

PO Box 9227

1 Medical Center Drive

Morgantown, WV 26506

Tel: 1-(304) 293-3683

Fax: 1-(304) 293-7105

Email: [jhollander@hsc.wvu.edu](mailto:jhollander@hsc.wvu.edu)

**Supplemental Figure Legends**

**Additional file 2: Figure S1. Mitochondrial bioenergetics of other tissues and organ systems.** **(A)** In young adult (n = 7 Sham, n = 5 Ex) animals state 3 and state 4 respiration was assessed using glucose (glutamine) and fatty-acid-mediated pathways (palmitoyl carnitine), normalized to number of mitochondria. **(B)** ETC complex activities for complex I in fetal, young adult, and maternal tissues. **(C)** ETC complex activities for complex III in fetal, young adult, and maternal tissues. **(D)** ETC complex activities for complex IV in fetal, young adult, and maternal tissues. **(E)** ETC complex activities for complex V (ATP synthase) in fetal, young adult, and maternal tissues. Sham = control filtered air exposed, Ex = nano-TiO_2_ exposed, Maternal (M) = 12-week old pregnant dams, Fetal (F) = GD (15), Young Adult (YA) = 11 weeks, P-Carnitine = palmitoyl carnitine. All data are presented as the mean ± standard error of the mean (SEM). * = *P* ≤ 0.05 for Ex vs. Sham.

**Additional file 2: Figure S2.** **ROS-mediated pathways in other tissues and organ systems.** **(A)** Hydrogen peroxide (H_2_O_2_) concentration in fetal, young adult, and maternal tissues normalized to protein content. **(B)** Hif1α activity was measured in fetal and maternal tissues and normalized to protein content. **(C)** Activity of DNA methyltransferases (Dnmts) in fetal (n = 4 Sham, n = 5 Ex) heart tissue, normalized to protein content. **(D)** Dnmt3b expression levels were assessed in young adult (n = 3 Sham, n = 3 Ex) animals and normalized using anti-Gapdh primary antibody. **(E)** Global 5-mC DNA methylation levels were evaluated in young adult (n = 7 Sham, n = 5 Ex) hearts, normalized to DNA concentration. Sham = control filtered air exposed, Ex = nano-TiO_2_ exposed, Maternal (M) = 12-week old pregnant dams, Fetal (F) = GD (15), Young Adult (YA) = 11 weeks, Hif1α = Hypoxia-inducible factor 1-alpha. Dnmt3b = DNA (cytosine-5)-methyltransferase 3b. All data are presented as the mean ± standard error of the mean (SEM). * = *P* ≤ 0.05, ** = *P* ≤ 0.01 for Ex vs. Sham.

**Additional file 2: Figure S1. Bioenergetics**


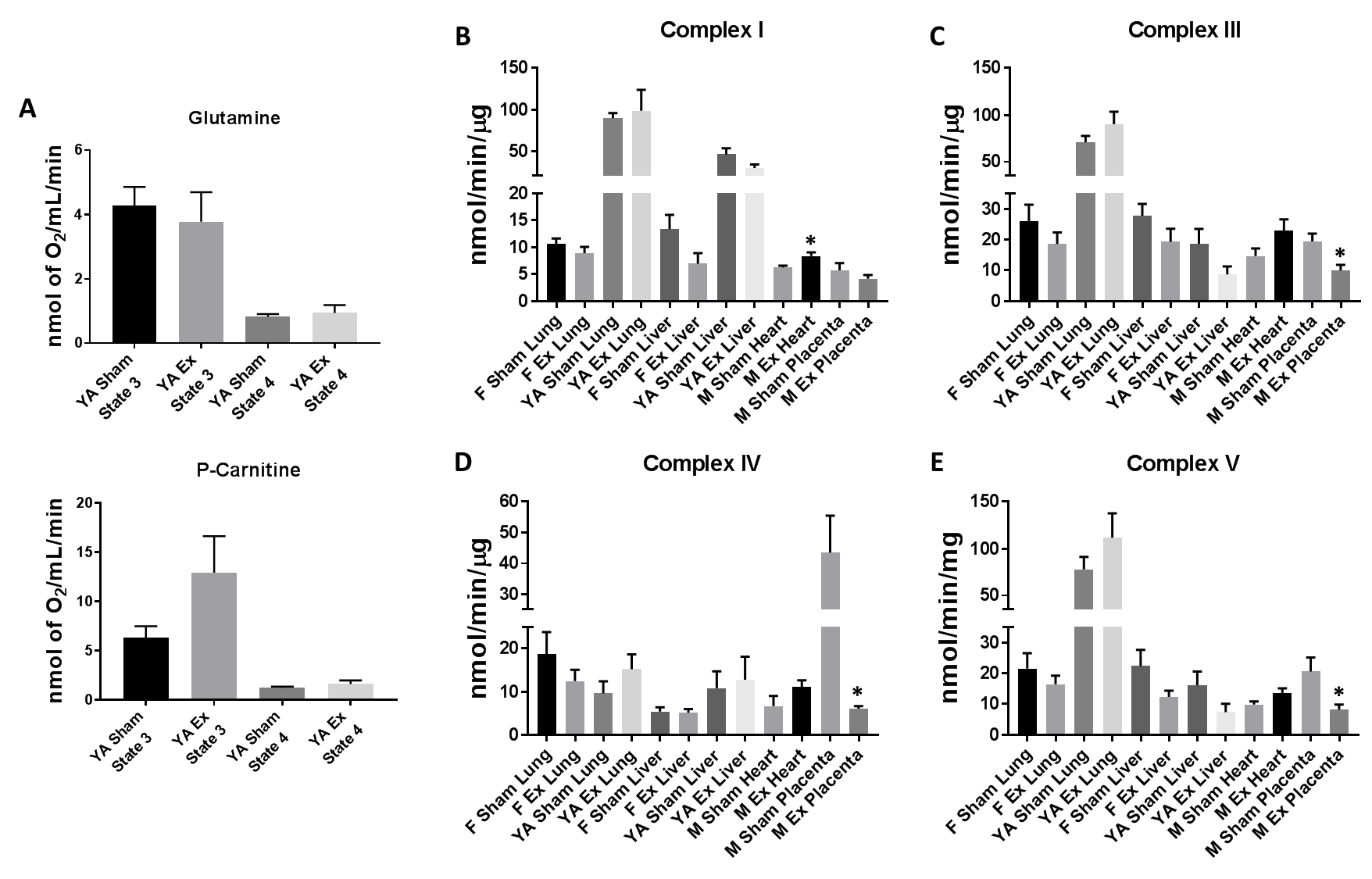


**Additional file 2: Figure S2. ROS-Mediated Effects**


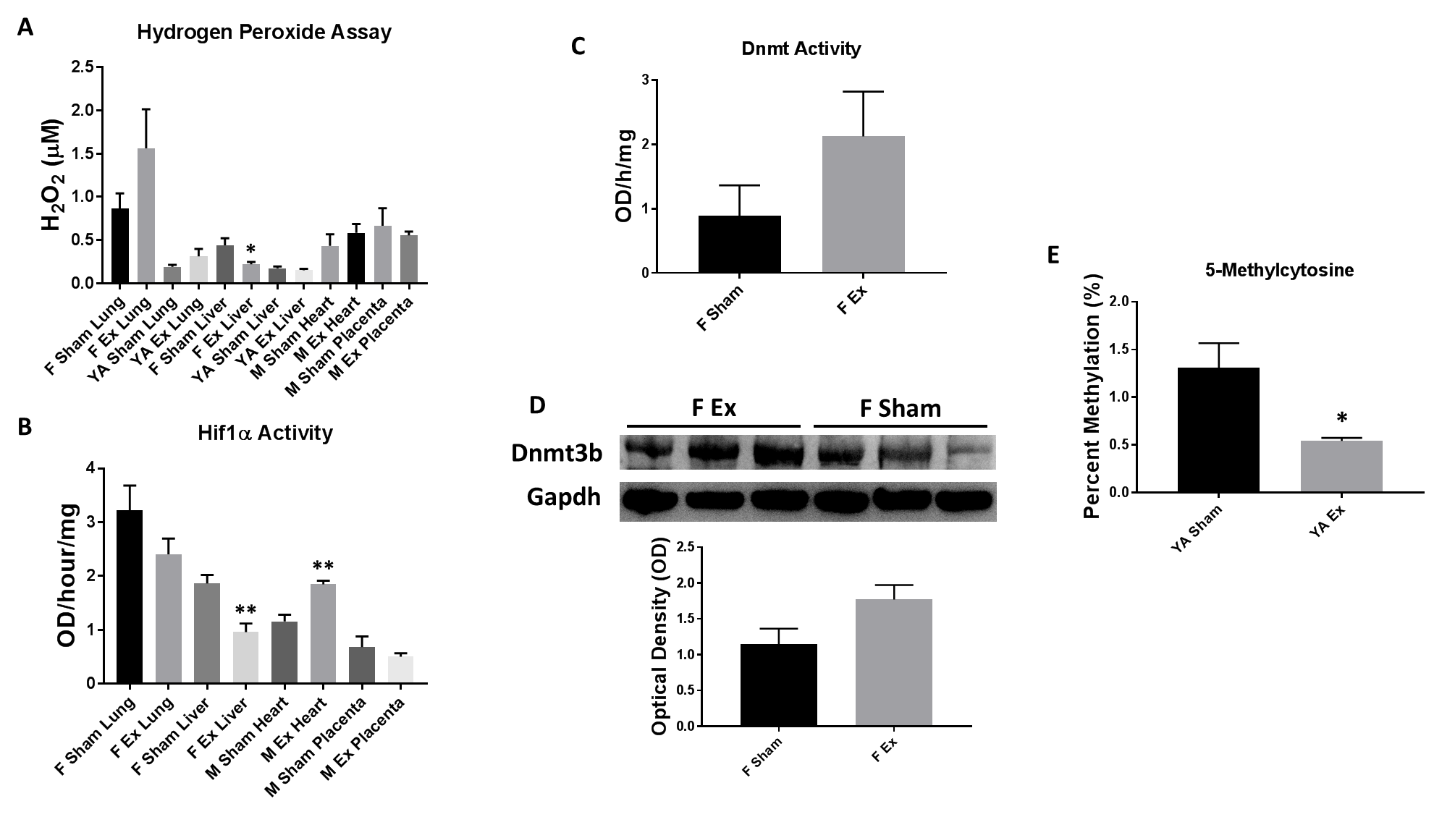

Supplement: Supplementary file 2 — Supplemental figures to the primary manuscript including, mitochondrial bioenergetics of other tissues and the assessment of ROS mediated pathways in other tissues and organ systems in maternal, fetal, and young adult animals. These additional parameters are provided in order to ensure a thorough assessment and further insight into the ROS-related consequences of maternal nano-TiO2 inhalation exposure during gestation. (DOCX 267 kb) [file 12989_2019_310_MOESM2_ESM.docx]
